# Tumor suppressor function of SHMT in a *Drosophila Ras*^*V12*^*Dlg*^*RNAi*^ model: DNA damage and synergistic gene-nutrient interaction with PLP

**DOI:** 10.1038/s41419-026-08602-7

**Published:** 2026-03-26

**Authors:** Chiara Angioli, Angelo Ferriero, Eleonora Pilesi, Giulia Tesoriere, Beatrice Agostini, Angela Tramonti, Roberto Contestabile, Fiammetta Vernì

**Affiliations:** 1 Dept. of Biology and Biotechnology “Charles Darwin”, Sapienza University of Rome, Rome, Italy; 2 Institute of Molecular Biology and Pathology, National Research Council, Rome, Italy; 3 Department of Biochemical Sciences “A. Rossi Fanelli”, Sapienza, University of Rome, Rome, Italy; 4 Istituto Pasteur-Fondazione Cenci Bolognetti, Sapienza, University of Rome, Rome, Italy

**Keywords:** Cancer, Diseases

## Abstract

Serine hydroxymethyltransferase (SHMT) is a key enzyme in one-carbon (1 C) metabolism, essential for nucleotide synthesis and epigenetic maintenance. In mammals, there are two distinct SHMT isozymes: the cytosolic SHMT1 and the mitochondrial SHMT2. Several studies report that high SHMT levels in cancer contribute to metabolic reprogramming. Conversely, a limited number of studies have linked decreased SHMT1 expression to the progression and poor prognosis of hepatocellular carcinoma and renal cell carcinoma, suggesting that SHMT may play dual roles as an oncogene or tumor suppressor, depending on the cellular context. However, the molecular mechanisms underlying SHMT tumor suppressor role remain unknown. In this work, we used the *Drosophila Ras*^*V12*^*Dlg*^*RNAi*^ cancer model to investigate the effects of SHMT depletion on cancer progression and the associated mechanisms. We found that RNAi-mediated *SHMT* silencing promotes the progression of *Ras*^*V12*^*Dlg*^*RNAi*^ cancers by impairing thymidylate biosynthesis in the folate pathway. *SHMT* depletion in *Ras*^*V12*^*Dlg*^*RNAi*^ cells causes DNA and chromosome damage and renders these cells sensitive to genotoxic stressors such as X-rays or hydroxyurea. Genome instability is correlated with cancer progression, and it is largely due to the generation of reactive oxygen species (ROS) and, to a lesser degree, to replicative stress and compromised DNA repair mechanisms, all arising from SHMT depletion. Antioxidant treatment with N-acetyl cysteine (NAC) significantly reduces both DNA damage and tumor progression. Intriguingly, the combined depletion of SHMT and its cofactor pyridoxal 5’-phosphate (PLP) further increases oxidative stress, leading to extensive DNA damage that induces apoptosis in *Ras*^*V12*^*Dlg*^*RNAi*^ cells, thereby limiting the tumor growth. Taken together, our data suggest that a diminished SHMT activity may drive the progression of *Ras*^*V12*^*Dlg*^*RNAi*^ cancers through ROS-induced genome instability. Additionally, our study points to a novel gene-nutrient interaction, SHMT-PLP, that impacts cancer growth with potential therapeutic implications.

## Introduction

The one carbon (1 C) metabolism is based on the activity of three interconnected pathways: the folate cycle, the methionine cycle, and the trans-sulfuration pathway [1]. In folate cycle the pyridoxal 5’-phosphate (PLP)-dependent enzyme serine hydroxymethyltransferase (SHMT, EC 2.1.2.1) catalyzes the reversible conversion of serine to glycine. This reaction concurrently transfers 1 C units to tetrahydrofolate (THF), yielding N5, N10-methylene THF [1]. This compound is then used for thymidylate (dTMP) synthesis by thymidylate synthase (TS or TYMS, EC 2.1.1.45). Alternatively, N5, N10-methylene THF can be reduced to methyl-THF which enters the methionine cycle to produce S-Adenosylmethionine (SAM or AdoMet), the major methyl group donor of the cell [2].

Two different SHMT genes exist in mammalian genomes: *SHMT1*, encoding the cytoplasmic isoform, and *SHMT2*, encoding the mitochondrial isoform [3]. Cytoplasmic SHMT1 enzyme regulates the de novo synthesis of purines and dTMP, and the remethylation of homocysteine to methionine. Mitochondrial SHMT2 produces 1 C units exported in the cytoplasm as formate to sustain the cytoplasmic 1 C metabolism. Thymidylate de novo synthesis takes place also in the nucleus supported by the activity of SUMOylated SHMT1, Dihydrofolate Reductase (DHFR), and TS proteins [3]. SHMT1 also behaves as an unconventional RNA binding protein able to control the translation of its own transcript [4]. Moreover, SHMT1 enzymatic activity is riboregulated by the 5′ untranslated region (5’-UTR) binding to the SHMT2 mRNA [5, 6].

1 C pathway due to its critical role in nucleotide synthesis and cellular methylation significantly contributes to the metabolic reprogramming that fuels cancer growth. SHMT enzymes are known for their involvement in various aspects of cancer biology. Consequently, targeted therapies against SHMT proteins have already been developed for the treatment of several cancer types [7]. Increased *SHMT2* expression has been related to the development and metastasis of breast cancer [8] and oral squamous cell carcinoma, where its strong expression correlates with poor prognosis [9]. *SHMT2* overexpression has also been observed in glioma [10], lymphoma [11], bladder [12, 13] and gastric cancer [14]. Furthermore, SHMT2 has been found to modulate metabolic reprogramming and epigenetics in papillary thyroid cancer [15]. Similarly, SHMT1 has also been shown to promote progression in various cancer types such as glioma [16], lung [17], and ovarian cancers [18].

Conversely, a smaller but growing body of research suggests that SHMT may act as a tumor suppressor in specific contexts, highlighting its complex and context-dependent roles in cancer biology. *SHMT1* is downregulated in both human hepatocellular carcinoma (HCC) [19] and renal cell carcinoma (RCC) [20]. This downregulation directly correlates with unfavorable clinicopathological features and a poorer patient prognosis, suggesting that SHMT1 may play a protective role when expressed at normal levels. In human HCC cells, SHMT1 depletion has been shown to promote epithelial-mesenchymal transition (EMT) [19], a critical process in cancer metastasis, indicating that SHMT1 may normally suppress the spread of cancer cells. In RCC, the transcription factor HOXD8 has been identified as regulator responsible for *SHMT1* downregulation [20]. Furthermore, *SHMT1* hemizygosity has been found to be correlated with an increased risk of intestinal tumor in *Apc* ^*min/+*^ mice [21].

Consistent with the tumor suppressor role of SHMT, polymorphic variants of the *SHMT1* gene, associated with reduced enzyme activity, have been linked to increased risk of several cancers, including breast [22], lung [23], and rectal cancer [24], as well as with adult acute lymphocytic leukemia [25] and malignant lymphoma [26]. However, many of these associations are based on studies with relatively small sample sizes and thus require further validation in larger cohorts.

The *Drosophila* genome has a single *SHMT* gene encoding alternative transcripts, producing both a cytoplasmic isoform and a mitochondrial isoform. Moreover, the fly SHMT enzyme, similarly to mammalian SHMT1, can localize to the nucleus to sustain dTMP biosynthesis [27]. We recently demonstrated that RNAi-mediated silencing of *SHMT* in *Drosophila* produces DNA damage hampered by a simultaneous reduced availability of its enzymatic cofactor, the pyridoxal 5’-phosphate, (PLP) which represents the catalytical active form of vitamin B6 [28]. This finding suggests that SHMT depletion may impact on cancer by compromising genome integrity and that this effect might be modulated by PLP availability. To test this hypothesis here we studied the tumor suppressor role of SHMT in *Drosophila* using a cancer model based on the expression of the oncogenic Ras^V12^ protein [29] combined with the silencing of *Disc large* (*Dlg*) polarity gene [30] with the aim of dissecting the underlying mechanisms.

## Results

### *SHMT* silencing promotes the progression of *Ras*^*V12*^*Dlg*^*RNAi*^ tumors through folate pathway

**Fig. 1:**
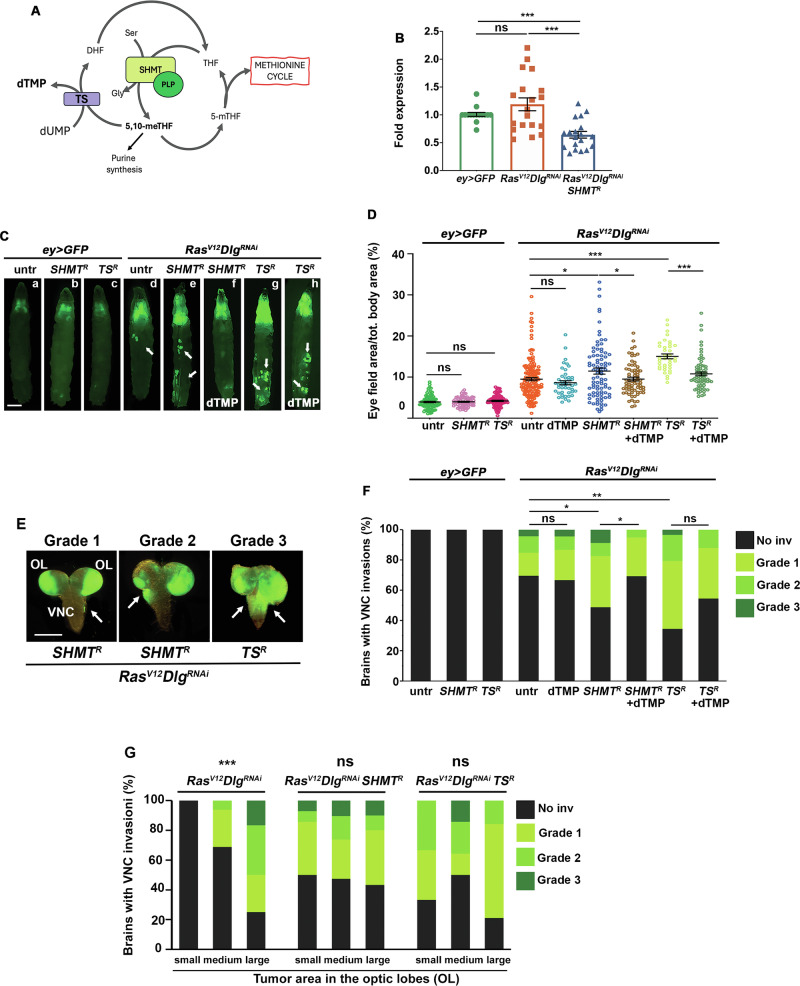
*SHMT* silencing promotes the progression of *Ras*^*V12*^*Dlg*^*RNAi*^ tumors. **A** Simplified scheme of folate pathway. SHMT in the presence of PLP cofactor produces 5,10-meTHF in turn used for purine synthesis and dTMP synthesis mediated by TS enzyme. The conversion of 5,10-meTHF into 5-mTHF produces one carbon units for methionine cycle. SHMT serine hydroxymethyltransferase, PLP pyridoxal 5’-phosphate, 5,10-meTHF 5,10-methylenetetrahydrofolate; 5-mTHF 5-methyltetrahydrofolate, DHF dihydrofolate. THF tetrahydrofolate, TS thymidylate synthase, dUMP deoxyuridine monophosphate; dTMP= deoxythymidine monophosphate, Ser serine, Gly glycine. **B** *SHMT* mRNA levels evaluated by RT-qPCR analysis. *SHMT* mRNA levels in *Ras*^*V12*^*Dlg*^*RNAi*^ eye discs are not significantly different from the levels found in *ey* > *GFP* control discs. The RNAi-mediated silencing of *SHMT* significantly decreases its expression levels. Error bars, SEM. ***P < 0.001 (unpaired *t*-test). Ns = not significant. *P* = 0.19. *SHMT*^*R*^ = *SHMT*^*RNAi*^. **C** Representative images of larvae showing GFP-labeled primary tumors. *ey* > *GFP Ras*^*V12*^*Dlg*^*RNAi*^ larvae (abbreviated as *Ras*^*V12*^*Dlg*^*RNAi*^) express the oncogenic *Ras*^*V12*^ in imaginal eye disc cells, along with a *UAS-Dlg* hairpin RNAi construct and a *UAS-GFP* construct. Scale bar, 0.5 mm. The RNAi-mediated silencing of *SHMT* (or *TS*) enhances the tumor growth (e, g). Tumors are rescued (or reduced) by dTMP supplementation (f, h). In *ey* > *GFP* control discs, only GFP is expressed. Secondary tumors far from cephalic area are indicated by arrows. dTMP=thymidylate. *TS*^*R*^ = *TS*^*RNAi*^. **D** Quantification of GFP-positive eye field area relative to total body area (%). Error bars, SEM. *P < 0.05; ***P < 0.001 (unpaired *t*-test). Ns = not significant *ey* > *GFP SHMT*^*R*^ vs *ey* > *GFP* *P* = 0.85; *ey* > *GFP TS*^*R*^ vs *ey* > *GFP* *P* = 0.26; *Ras*^*V12*^*Dlg*^*RNAi*^ dTMP vs *Ras*^*V12*^*Dlg*^*RNAi*^ P = 0.25. Number of scored larvae in at least three independent experiments*: ey* > *GFP n* = 98; *ey* > *GFP SHMT*^*R*^ *n* = 71; *ey* > *GFP TS*^*R*^ *n* = 78; *Ras*^*V12*^*Dlg*^*RNAi*^*n* = 152; *Ras*^*V12*^*Dlg*^*RNAi*^ dTMP *n* = 45; *Ras*^*V12*^*Dlg*^*RNAi*^ *SHMT*^*R*^ *n* = 85; *Ras*^*V12*^*Dlg*^*RNAi*^*SHMT*^*R*^ *dTMP n* = *69; Ras*^*V12*^*Dlg*^*RNAi*^*TS*^*R*^*n* = *37 Ras*^*V12*^*Dlg*^*RNAi*^*TS*^*R*^ dTMP *n* = 72. **E** Larval brains showing tumor invasions on the ventral nerve cord (VNC) were assigned to three different categories based on invasion degree. Grade1=mild; Grade 2=moderate; Grade 3=severe. Note that the *ey*-Flippase that generates GFP-marked *Ras*^*V12*^*Dlg*^*RNAi*^ cells is also active in optic lobes (OL). Scale bar, 100 µm. **F** Quantification of results. The green-labeled portion of each column represents the percentage of brains with VNC invasions. The black portion indicates the percentage of brains without invasions. The three different types of green represent arbitrary levels of invasion exemplified in (**E**). Brains were from larvae collected at 12–14 days after cross. Statistics were assessed by chi square test and refer to the percentage of total invasion phenotype. *P < 0.05; **P < 0.01. Ns = not significant. *Ras*^*V12*^*Dlg*^*RNAi*^ dTMP *vs Ras*^*V12*^*Dlg*^*RNAi*^ *P* = 0.76; *Ras*^*V12*^*Dlg*^*RNAi*^*TS*^*R*^ dTMP vs. *Ras*^*V12*^*Dlg*^*RNAi*^*TS*^*R*^ *P* = 0.11. Number of scored brains in at least three independent experiments: *ey* > *GFP n* = 40; *ey* > *GFP SHMT*^*R*^ *n* = 35; *ey* > *GFP TS*^*R*^ *n* = 38; *Ras*^*V12*^*Dlg*^*RNAi*^ *n* = 46; *Ras*^*V12*^*Dlg*^*RNAi*^ dTMP *n* = 45; *Ras*^*V12*^*Dlg*^*RNAi*^*SHMT*^*R*^ *n* = 80; *Ras*^*V12*^*Dlg*^*RNAi*^ *SHMT*^*R*^ dTMP *n* = 39*; Ras*^*V12*^*Dlg*^*RNAi*^*TS*^*R*^ *n* = *29; Ras*^*V12*^*Dlg*^*RNAi*^*TS*^*R*^ dTMP *n* = 33. **G** Relationship between brain optic lobe primary tumor size and grade of VNC invasions. Optic lobe tumors were grouped into three size categories based on the percentage of the GFP-labeled area: small (30–50%), medium (51–70%), and large (71–100%). The three different types of green represent arbitrary levels of invasion exemplified in the (**E**). Chi-square test of independence was used to compare the distribution of invasion grades across the tumor size categories. Ns=not significant *Ras*^*V12*^*Dlg*^*RNAi*^*SHMT*^*R*^ *P* = 0.97; *Ras*^*V12*^*Dlg*^*RNAi*^*TS*^*R*^ *P* = 0.08. Number of scored brains: *Ras*^*V12*^*Dlg*^*RNAi*^ *n* = 38; *Ras*^*V12*^*Dlg*^*RNAi*^*SHMT*^*R*^ *n* = 63; *Ras*^*V12*^*Dlg*^*RNAi*^*TS*^*R*^ *n* = 39.

SHMT plays a key role in the folate pathway, enabling the synthesis of purines and pyrimidines (Fig. 1A). To investigate its tumor suppressor role in *Drosophila*, we tested the effect of *SHMT* silencing on progression of *ey* > *GFP Ras*^*V12*^*Dlg*^*RNAi*^ tumors (hereinafter *Ras*^*V12*^*Dlg*^*RNAi*^). This malignant cancer model is established in eye antennal disc cells through the concomitant overexpression of *Ras*^*V12*^ oncogene [29] and the RNAi-mediated silencing of *Disc large (Dlg)* polarity gene [30]. Concurrent green fluorescent protein (GFP) expression in these eye discs enables visualization of tumor expansion and dissemination [31–33].

Since many human tumors express SHMT at high levels, we first ensured that *SHMT* was expressed at wild-type levels in eye discs from *Ras*^*V12*^*Dlg*^*RNAi*^ larvae (Fig. 1B). To reduce the function of SHMT in the *Ras*^*V12*^*Dlg*^*RNAi*^ eye discs we used the RNA interference (VDRC line *SHMT*^*v19206*^) which was effective in decreasing the *SHMT* expression by approximately 40% (Fig. 1B) and the catalytic activity by about 75% (Figure S1). The presence of *Ras*^*V12*^*Dlg*^*RNAi*^ tumors in eye discs results in a developmental delay, leading larvae to reach the third instar 12–14 days after the cross. Consistently, in all experiments we examined tumor-bearing larvae collected in this interval of time.

*SHMT* silencing significantly increased the GFP-labeled cephalic area in *Ras*^*V12*^*Dlg*^*RNAi*^*SHMT*^*RNAi*^ larvae compared to *Ras*^*V12*^*Dlg*^*RNAi*^ controls (11% of total body vs 9%), (Fig. 1C, D, Figure S2). Conversely, it did not influence the GFP expression in non-tumoral control eye discs (*ey* > *GFP SHMT*^*RNAi*^) (Fig. 1C, D).

*SHMT* silencing also increased the invasiveness of *Ras*^*V12*^*Dlg*^*RNAi*^ tumors by enhancing secondary tumor formation, which was quantified in larval brains by assessing the frequency of invasions on the ventral nerve cord (VNC) [33, 34] (Fig. 1E, F). SHMT depletion promoted invasions in approximately 50% of brains (vs. about 30% in *Ras*^*V12*^*Dlg*^*RNAi*^ controls) (Fig. 1F), indicating that reducing SHMT activity also exacerbates the metastatic potential of *Ras*^*V12*^*Dlg*^*RNAi*^ tumors. Remarkably, the severity of VNC invasion did not correlate statistically with the size of the primary tumors on optic lobes (Fig. 1G). This suggests that SHMT depletion confers an intrinsic metastatic capacity, thus ruling out the possibility that the increase in metastasis was simply due to a higher number of primary tumor cells. Furthermore, the extent of tumor invasion showed no correlation with larval age (Figure S3).

The enzyme thymidylate synthase (TS), positioned downstream of SHMT (Fig. 1A), uses N5, N10-methylene-THF (generated by SHMT) as a substrate for the conversion of dUMP to dTMP. *TS* depletion via RNAi (confirmed by RT-qPCR, Figure S4) increased primary (15% of total body area) and secondary tumor formation (65% of brains with invasions) in *Ras*^*V12*^*Dlg*^*RNAi*^ larvae (Fig. 1C–G; Figure S3). Thymidylate (dTMP) supplementation in *Ras*^*V12*^*Dlg*^*RNAi*^ *SHMT*^*RNAi*^ larvae rescued tumor phenotypes, restoring tumor progression to baseline levels observed in *Ras*^*V12*^*Dlg*^*RNAi*^ controls (Fig. 1C, D, F; Figure S5). In *Ras*^*V12*^*Dlg*^*RNAi*^*TS*^*RNAi*^ larvae dTMP supplementation resulted in a significant reduction in primary tumor size (10.78% of the body area). Additionally, the proportion of invaded brains decreased from 65.5% to 45%, although this reduction did not reach statistical significance (Fig. 1C, D, F).

The same effect as SHMT depletion on *Ras*^*V12*^*Dlg*^*RNAi*^ tumors was produced by the SHMT inhibitor metformin [35] (Figure S6).

Notably, reduced SHMT activity can also affect the development of *Ras*^*V12*^*csk*^*-/-*^ tumors [36] (Figure S6), while it did not affect the transformation of *Ras*^*V12*^ benign tumors in aggressive forms [29] (Figure S6), suggesting that SHMT vulnerability may be preferentially acquired when *Ras*^*V12*^ cooperates with a second oncogenic lesion.

### SHMT depletion induces genome instability in *Ras*^*V12*^*Dlg*^*RNAi*^ eye discs

**Fig. 2:**
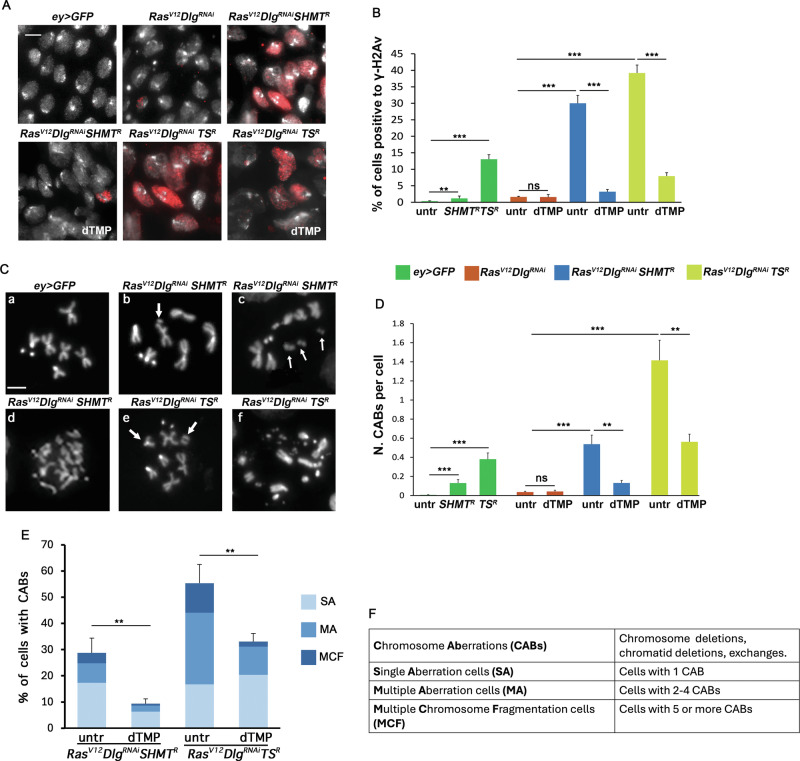
RNAi-mediated *SHMT* silencing causes DNA and chromosome damage in *Ras*^*V12*^*Dlg*^*RNAi*^ eye discs. **A** Examples of eye disc nuclei positive γ-H2Av antibody (red). Scale bar, 5 µm. **B** Quantification of results. Error bars, SEM. **P < 0.01; ***P < 0.001 (unpaired *t*-test). Ns = not significant *P* = 0.839. Total number of examined cells in at least three independent experiments: *ey* > *GFP n* = 1867; *ey* > *GFP SHMT*^*R*^ *n* = 5186; *ey* > *GFP TS*^*R*^ *n* = 4009; *Ras*^*V12*^*Dlg*^*RNAi*^ *n* = 5092*; Ras*^*V12*^*Dlg*^*RNAi*^ dTMP *n* = 2918; *Ras*^*V12*^*Dlg*^*RNAi*^*SHMT*^*R*^ *n* = 2461; *Ras*^*V12*^*Dlg*^*RNAi*^*SHMT*^*R*^ dTMP *n* = 5431; *Ras*^*V12*^*Dlg*^*RNAi*^*TS*^*R*^ *n* = 3058; *Ras*^*V12*^*Dlg*^*RNAi*^*TS*^*R*^ dTMP *n* = 3295. **C** Examples of chromosome aberrations (CABs) in metaphases of eye discs of indicated genotypes. (**a**) wild type female metaphase; (**b**) chromatid deletion of a major autosome (arrow); (**c**) metaphase with multiple breaks (arrowed); (**d**) metaphase with fragmented chromosomes; (**e**) metaphase with centric deletion (arrows); (**f**) metaphase with severely fragmented chromosomes. Scale bar, 5 µm. **D** Quantification of results. Error bars, SEM. **P < 0.01; ***P < 0.001 (unpaired *t*-test). Ns = not significant, *P* = 0.07. Total number of examined cells in at least three independent experiments: *ey* > *GFP n* = 542 (13 discs); *ey* > *GFP SHMT*^*R*^ *n* = 446 (9 discs); *ey* > *GFP TS*^*R*^ *n* = 491 (10 discs); *Ras*^*V12*^*Dlg*^*RNAi*^*n* = 538 (8 discs); *Ras*^*V12*^*Dlg*^*RNAi*^ dTMP *n* = 471 (7 discs); *Ras*^*V12*^*Dlg*^*RNAi*^*SHMT*^*R*^ *n* = 501 (16 discs); *Ras*^*V12*^*Dlg*^*RNAi*^*SHMT*^*R*^ dTMP *n* = 726 (7 discs); *Ras*^*V12*^*Dlg*^*RNAi*^*TS*^*R*^ *n* = 274 (10 discs); *Ras*^*V12*^*Dlg*^*RNAi*^*TS*^*R*^ dTMP *n* = 309 (11 discs). **E** Percentage of cells with CABs. Error bars, SEM (calculated on the total frequency of cells with CABs). **P < 0.01 (unpaired *t*-test). SA single aberration cells (panel **C**, b, e); MA multiple aberration cells (**C**); MCF multiple chromosome fragmentation cells (panel **C**, d, f). **F** List of acronyms and corresponding definitions used to categorize metaphase cells based on the number of chromosome aberrations.

Our previous finding that *SHMT* silencing induces genome instability in *Drosophila* neuroblasts [28] suggested that DNA damage may be involved in the progression of *Ras*^*V12*^*Dlg*^*RNAi*^*SHMT*^*RNAi*^ tumors. Consistently, we found that *SHMT* depletion in *Ras*^*V12*^*Dlg*^*RNAi*^ cells increased the accumulation of γ-H2Av foci, a marker of DNA double strand breaks (DSBs) (31% of positive cells *vs* 1.5% in *Ras*^*V12*^*Dlg*^*RNAi*^), and this effect was completely rescued by dTMP supplementation (3.2%) (Fig. 2A, B). Similarly, *TS* depletion in *Ras*^*V12*^*Dlg*^*RNAi*^ cells also increased the frequency of γ-H2Av foci (39.2%) and dTMP significantly decreased this percentage (7.95%) (Fig. 2B).

It is known that unrepaired or malrepaired DSBs can result in chromosome aberrations (CABs) [37] that are in turn associated with cancer [38]. To assess chromosome damage, we evaluated both the average number of CABs per cell and the frequency of affected cells. Consistently, eye discs from *Ras*^*V12*^*Dlg*^*RNAi*^*SHMT*^*RNAi*^ larvae displayed 0.54 CABs per cell (vs 0.035 in *Ras*^*V12*^*Dlg*^*RNAi*^) (Fig. 2C, D) and 28% of cells with CABs (Fig. 2E, F). Of these, 17% showed single breaks (Single Aberration cells, SA), 7% displayed 2 to 4 breaks (Multiple Aberration cells, MA), and 4% presented 5 or more breaks (Multiple Chromosome Fragmentation cells, MCF). This last class also includes cells with severely multi-fragmented chromosomes. dTMP administration significantly decreased the chromosome damage (0.13 CABs per cell and 9% of cells with CABs) (Fig. 2D, E).

*TS* silencing resulted in a very high frequency of CABs in *Ras*^*V12*^*Dlg*^*RNAi*^ tumor eye discs (1.4 CABs per cell and 56% of cells with CABs), and dTMP supplementation significantly reduced the mean number of CABs per cell to 0.56 CABs per cell and the percentage of cells with CABs to 33% (Fig. 2D, E).

### *Ras*^*V12*^*Dlg*^*RNAi*^*SHMT*^*RNAi*^ eye discs are sensitive to genotoxic stressors

Given that SHMT regulates purine and pyrimidine synthesis, it is conceivable that DNA damage may result from impaired DNA repair and/or synthesis caused by an unbalanced nucleotide pool [39]. Additionally, DNA damage might originate from increased levels of reactive oxygen species (ROS) whose production has been shown to increase following SHMT depletion [19, 33, 40]. Thus, we addressed these issues.

**Fig. 3:**
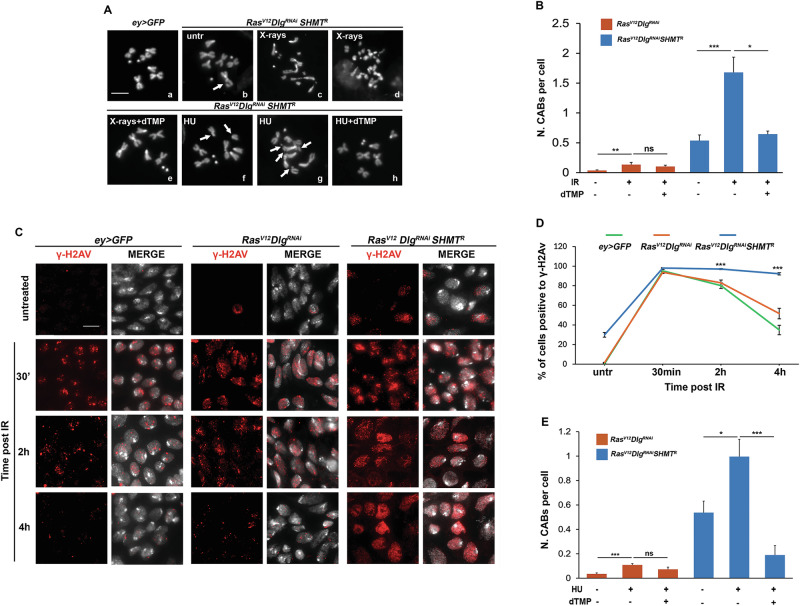
*SHMT* silencing induces X-ray and HU sensitivity in *Ras*^*V12*^*Dlg*^*RNAi*^ cells. **A** Examples of CABs in eye discs of indicated genotypes/treatments. (a) wild type female metaphase; (b) chromatid deletion of a major autosome (arrowed); (c,d) metaphases with multiply fragmented chromosome; (e, h) wild type metaphases; (f) centric deletion of a major autosome (arrows); (g) metaphase with multiply fragmented chromosomes. Scale bar, 5 µm. dTMP= deoxythymidine monophosphate; HU hydroxyurea (1 mM). **B** Quantification of X-ray treatment. Error bars, SEM. *P < 0.05; **P < 0.01; ***P < 0.001 (unpaired *t*-test). Ns=not significant *P* = 0.06. IR = X-ray treatment (2.5 Gy). Total number of examined cells in at least three independent experiments: untreated *Ras*^*V12*^*Dlg*^*RNAi*^ *n* = 538 (8 discs); X-ray-treated-*Ras*^*V12*^*Dlg*^*RNAi*^ *n* = 253 (6 discs); X-ray+dTMP-treated *Ras*^*V12*^*Dlg*^*RNAi*^ *n* = 194 (5 discs); untreated *Ras*^*V12*^*Dlg*^*RNAi*^*SHMT*^*R*^ *n* = 501 (16 discs); X-ray-treated *Ras*^*V12*^*Dlg*^*RNAi*^*SHMT*^*R*^ *n* = 125 (7 discs); X-ray+dTMP-treated *Ras*^*V12*^*Dlg*^*RNAi*^*SHMT*^*R*^ *n* = 166 (5 discs). **C** Dissolution kinetics of X-ray-induced (5 Gy) γ-H2Av foci (red) in nuclei of larval eye discs of indicated genotypes. Untr=untreated. A high proportion of γ-H2Av-positive *Ras*^*V12*^*Dlg*^*RNAi*^*SHMT*^*R*^ cells persists at 4 hours post-irradiation (PIR). Scale bar, 5 µm. **D** Quantification of γ-H2Av foci resolution. Each time point post-irradiation represents the mean value from three independent experiments ± SEM. Statistical analysis (unpaired *t*-test) reveled significant differences (***p < 0.001) between *Ras*^*V12*^*Dlg*^*RNAi*^*SHMT*^*R*^ cells and both *ey* > *GFP* and *Ras*^*V12*^*Dlg*^*RNAi*^ cells at 2 and 4 h PIR. At 4 h PIR the difference between *ey* > *GFP* and *Ras*^*V12*^*Dlg*^*RNAi*^ cells was not significant (*P* = 0.08). Total number of nuclei examined across at least three independent experiments: *ey* > *GFP n* = 1867; *ey* > *GFP* 30 min *n* = 2837*; ey* > *GFP* 2 h *n* = 1832; *ey* > *GFP* 4 h *n* = 2614; *Ras*^*V12*^*Dlg*^*RNAi*^ *n* = 5092; *Ras*^*V12*^*Dlg*^*RNAi*^ 30 min *n* = 4414; *Ras*^*V12*^*Dlg*^*RNAi*^ 2 h *n* = 2235; *Ras*^*V12*^*Dlg*^*RNAi*^ 4 h *n* = 2668*; Ras*^*V12*^*Dlg*^*RNAi*^ *SHMT*^*R*^ untr *n* = 2461; *Ras*^*V12*^*Dlg*^*RNAi*^ *SHMT*^*R*^ 30 min *n* = 3841; *Ras*^*V12*^*Dlg*^*RNAi*^*SHMT*^*R*^ 2 h *n* = 2415; *Ras*^*V12*^*Dlg*^*RNAi*^*SHMT*^*R*^ 4 h *n* = 3539. **E** Quantification of HU treatment (1 mM). Error bars, SEM. *P < 0.05; ***P < 0.001 (unpaired *t*-test). Ns=not significant P = 0.40. Total number of examined cells in at least three independent experiments: untreated *Ras*^*V12*^*Dlg*^*RNAi*^ *n* = 538 (8 discs); HU-treated *Ras*^*V12*^*Dlg*^*RNAi*^ *n* = 230 (7 discs); HU+dTMP-treated *Ras*^*V12*^*Dlg*^*RNAi*^ *n* = 797 (12 discs); untreated *Ras*^*V12*^*Dlg*^*RNAi*^*SHMT*^*R*^ *n* = 501 (16 discs); HU-treated *Ras*^*V12*^*Dlg*^*RNAi*^*SHMT*^*R*^ *n* = 238 (6 discs); HU + dTMP-treated-*Ras*^*V12*^*Dlg*^*RNAi*^*SHMT*^*R*^ *n* = 686 (12 discs).

To investigate the role of DNA repair, we performed an X-ray sensitivity test. We found that eye discs from *Ras*^*V12*^*Dlg*^*RNAi*^*SHMT*^*RNAi*^ larvae treated with 2.5 Gy of X-rays displayed a significantly increased frequency of CABs with an average of 1.68 CABs per cell, compared to 0.54 CABs per cell in untreated larvae (Fig. 3A, B). Notably, this observed frequency exceeds the additive value of 0.67 CABs per cell, derived from the sum of CAB frequency in untreated *Ras*^*V12*^*Dlg*^*RNAi*^ *SHMT*^*RNAi*^ cells (0.54) and X-ray treated *Ras*^*V12*^*Dlg*^*RNAi*^ cells (0.13), an outcome that would be expected if X-rays and SHMT silencing acted independently. Therefore, this non-additive increase suggests that SHMT depletion renders *Ras*^*V12*^*Dlg*^*RNAi*^ cells sensitive to X-rays, thereby implicating SHMT in the DNA repair process. Interestingly, dTMP supplementation attenuated the X-rays-induced damage, reducing CAB frequency to 0.64 CABs per cell (Fig. 3A, B).

To deeply investigate the involvement of SHMT in DNA repair, we monitored the dissolution of γ-H2Av repair foci over time following irradiation (Fig. 3C, D) [41]. Approximately 95% of *ey* > *GFP* control cells treated with 5 Gy of X-rays showed positive γ-H2Av immunostaining at 30 minutes post-irradiation (PIR). This percentage decreased to about 80% at 2 h PIR and further to 30% at 4 h PIR. We observed a similar trend in *Ras*^*V12*^*Dlg*^*RNAi*^ cells. In contrast, *SHMT* silencing in *Ras*^*V12*^*Dlg*^*RNAi*^ cells impaired foci dissolution, with 97% of cells remaining positive for γ-H2Av foci at 2 h PIR, and 92% still showed persistent foci at 4 h PIR. These findings suggest that SHMT depletion leads to delayed or defective DNA repair, which may contribute to the observed genomic instability.

Additionally, we assessed the sensitivity of *Ras*^*V12*^*Dlg*^*RNAi*^*SHMT*^*RNAi*^ eye discs to hydroxyurea (HU), a strong inhibitor of ribonucleotide reductase [42] that impairs DNA replication efficiency (Fig. 3A, E, Figure S7). Treatment with 1 mM HU resulted in an approximately two-fold increase in chromosome damage in *Ras*^*V12*^*Dlg*^*RNAi*^*SHMT*^*RNAi*^ eye discs (0.99 CABs per cell, compared to 0.54 in untreated cells)(Fig. 3A, E). This level of damage exceeded the sum of CAB frequencies (equal to 0.66) observed in untreated *Ras*^*V12*^*Dlg*^*RNAi*^*SHMT*^*RNAi*^ discs (0.54) and HU-treated *Ras*^*V12*^*Dlg*^*RNAi*^ cells (0.12). This non-additive increase indicates that SHMT-depleted cells are hypersensitive to HU-induced replication stress. Notably, dTMP supplementation rescued this phenotype. This suggests that the replicative stress may also contribute to DNA damage in the absence of SHMT.

### *SHMT* depletion causes ROS increase in *Ras*^*V12*^*Dlg*^*RNAi*^ cells

**Fig. 4:**
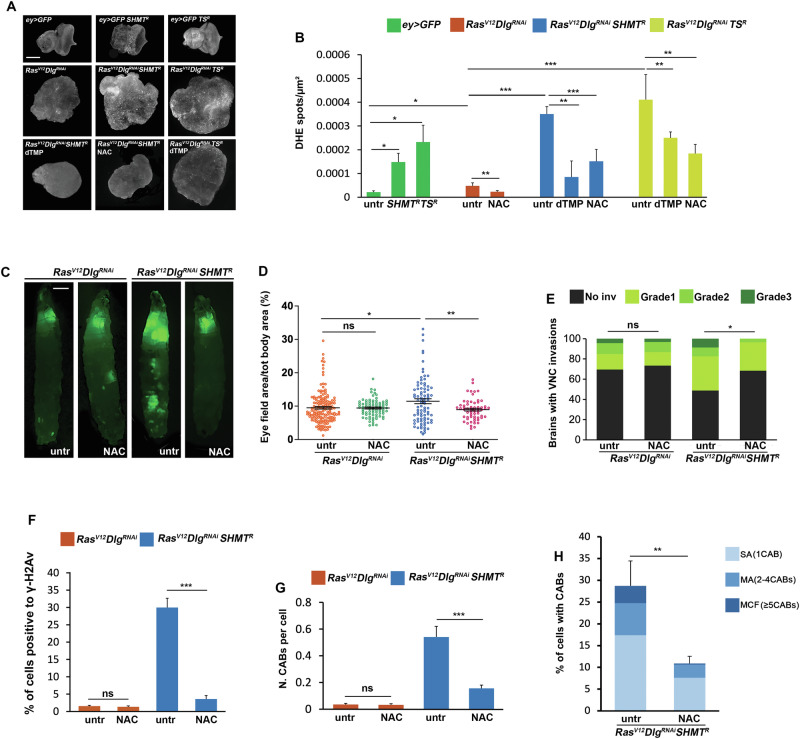
The role of ROS in *Ras*^*V12*^*Dlg*^*RNAi*^*SHMT*^*RNAi*^ cancer progression. **A** ROS accumulation in eye discs of indicated genotypes and treatments detected by dihydroethidium (DHE) staining. Scale bar, 20 μm. **B** Quantification of results obtained in three independent experiments. Spot density expressed as the number of DHE-positive spots per square micrometer (μm^2^). Error bars, SEM. *P < 0.05; **P < 0.01; ***P < 0.001 (unpaired *t*-test). NAC = N-acetyl cysteine (1 mg/ml). Number of scored eye discs*: ey* > *GFP n* = 8; *ey* > *GFP SHMT*^*R*^ *n* = 20; *ey* > *GFP TS*^*R*^ *n* = 18; *Ras*^*V12*^*Dlg*^*RNAi*^ *n* = 14; *Ras*^*V12*^*Dlg*^*RNAi*^ NAC *n* = 13; *Ras*^*V12*^*Dlg*^*RNAi*^*SHMT*^*R*^ *n* = 16 discs; *Ras*^*V12*^*Dlg*^*RNAi*^*SHMT*^*R*^ dTMP *n* = 8; *Ras*^*V12*^*Dlg*^*RNAi*^*SHMT*^*R*^ NAC *n* = 17; *Ras*^*V12*^*Dlg*^*RNAi*^*TS*^*R*^ *n* = 18; *Ras*^*V12*^*Dlg*^*RNAi*^*TS*^*R*^ dTMP *n* = 17; *Ras*^*V12*^*Dlg*^*RNAi*^*TS*^*R*^ NAC *n* = 12. **C** Representative images of *Ras*^*V12*^*Dlg*^*RNAi*^*SHMT*^*R*^ larvae in which NAC treatment rescues primary tumors. NAC = 1 mg/ml. Scale bar, 0.5 mm. **D** Quantification of results. GFP-positive eye field area relative to total body area. Error bars, SEM. *P < 0.05; **P < 0.01(unpaired *t*-test). Ns not significant *P* = 0.90. Number of larvae scored in at least three independent experiments: *Ras*^*V12*^*Dlg*^*RNAi*^*n* = 152; *Ras*^*V12*^*Dlg*^*RNAi*^ NAC *n* = 80; *Ras*^*V12*^*Dlg*^*RNAi*^*SHMT*^*R*^ *n* = 85; *Ras*^*V12*^*Dlg*^*RNAi*^*SHMT*^*R*^ NAC *n* = 60. **E** NAC treatment rescues secondary tumors in *Ras*^*V12*^*Dlg*^*RNAi*^*SHMT*^*R*^ larvae, quantification. NAC = 1 mg/ml. The green-labeled portion of each column represents the percentage of brains with VNC invasions. The black portion indicates the percentage of brains without invasions. The three different types of green represent arbitrary levels of invasions. Statistics were assessed by chi square test and refer to the percentage of total invasion phenotype *P < 0.01. Ns=not significant P = 0.67. Number of brains scored in at least three independent experiments*: Ras*^*V12*^*Dlg*^*RNAi*^*n* = 46*; Ras*^*V12*^*Dlg*^*RNAi*^ NAC *n* = 60; *Ras*^*V12*^*Dlg*^*RNAi*^*SHMT*^*R*^ *n* = 80; *Ras*^*V12*^*Dlg*^*RNAi*^*SHMT*^*R*^ NAC *n* = 57. **F** Percentage of cells positive to γ-H2Av antibody (red) in eye discs from larvae treated with 1 mg/ml NAC. Error bars, SEM. ***P < 0.001(unpaired *t*-test). Ns not significant, *P* = 0.76. Total number of examined cells in at least three independent experiments: *Ras*^*V12*^*Dlg*^*RNAi*^ *n* = 5092; *Ras*^*V12*^*Dlg*^*RNAi*^ NAC *n* = 4500; *Ras*^*V12*^*Dlg*^*RNAi*^*SHMT*^*R*^ *n* = 2461; *Ras*^*V12*^*Dlg*^*RNAi*^*SHMT*^*R*^ NAC *n* = 3389. **G** Number of CABs per cell in eye discs from larvae treated with 1 mg/ml NAC. Error bars, SEM ***P < 0.001 (unpaired *t*-test). Ns not significant *P* = 0.61. Number of examined cells in at least three independent experiments: *Ras*^*V12*^*Dlg*^*RNAi*^ *n* = 538 (8 discs); *Ras*^*V12*^*Dlg*^*RNAi*^ NAC *n* = 514 (7 discs)*; Ras*^*V12*^*Dlg*^*RNAi*^*SHMT*^*R*^ *n* = 501 (16 discs); *Ras*^*V12*^*Dlg*^*RNAi*^*SHMT*^*R*^ NAC n = 394 (8 discs). **H** Percentage of cells with CABs in eye discs from larvae treated with 1 mg/ml NAC. Error bars, SEM **P < 0.01 (unpaired *t*-test). Number of examined cells in three independent experiments: *Ras*^*V12*^*Dlg*^*RNAi*^*SHMT*^*R*^ *n* = 501 (16 discs); *Ras*^*V12*^*Dlg*^*RNAi*^*SHMT*^*R*^ NAC *n* = 394 (8 discs).

Consistent with previous findings that impaired folate pathway leads to ROS accumulation [19, 40], we observed that *Ras*^*V12*^*Dlg*^*RNAi*^ *SHMT*^*RNAi*^ eye discs exhibited a higher frequency of ROS compared to *Ras*^*V12*^*Dlg*^*RNAi*^ discs, as indicated by dihydroethidium (DHE) staining (Fig. 4A, B). In contrast ROS accumulation was reversed by dTMP supplementation (Fig. 4A, B). Similarly, TS depletion increased ROS levels in *Ras*^*V12*^*Dlg*^*RNAi*^ cells (Fig. 4A, B).

Since *Ras*^*V12*^*Dlg*^*RNAi*^*SHMT*^*RNAi*^ cells showed ROS accumulation, defective DNA synthesis and impaired DNA repair, we sought to evaluate the relative contributions of these factors to tumor progression. To this end we treated *Ras*^*V12*^*Dlg*^*RNAi*^*SHMT*^*RNAi*^ eye discs with the antioxidant N-acetyl cysteine (NAC) [43] and found that this treatment effectively rescued both ROS accumulation (Fig. 4A, B) and tumor progression features (Fig. 4C–E; Figure S5), thus providing strong evidence that ROS is a main causal factor for cancer progression.

**Fig. 5:**
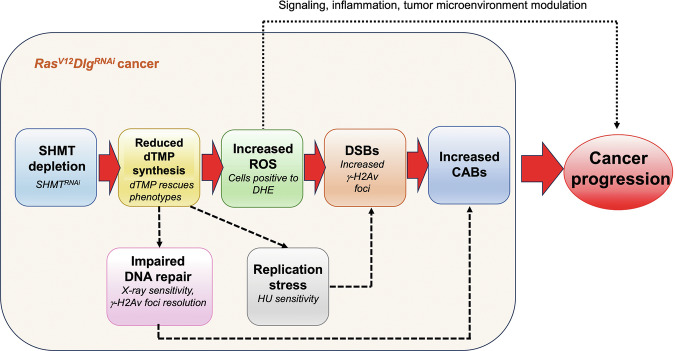
Scheme illustrating the possible mechanisms underlying the tumor suppressor role of SHMT on *Ras*^*V12*^*Dlg*^*RNAi*^ cancers. Primary pathway (red arrows): The depletion of SHMT causes a decreased dTMP synthesis, leading to an accumulation of ROS. High ROS levels generate DSBs, as evidenced by the increase of γ-H2Av foci. These DSBs constitute a critical factor contributing to CABs, which ultimately drives cancer progression. The recovery of tumors and DNA damage following NAC treatment highlights the central role of this pathway and points to ROS acting specifically via DNA damage. However, the contribution of other ROS-dependent mechanisms cannot be ruled out (black dotted line). Based on our data, additional mechanisms may operate as minor secondary pathways (black dashed arrows). Reduced dTMP synthesis also contributes to replication stress (evidenced by HU sensitivity) and impaired DNA repair mechanisms (evidenced by X-ray sensitivity and impaired γ-H2Av foci resolution). Replication stress may lead to DSBs; impaired DNA repair mechanisms may amplify the generation of CABs from DSBs.

Intriguingly, NAC treatment (1 mg/mL) significantly reduced the percentage of cells positive to γ-H2Av foci (from 31% to 3.57%) (Fig. 4F), the mean number of CABs per cell (from 0.54 to 0.15) (Fig. 4G) and the percentage of cells with chromosomal aberrations (from 28 to 13%) (Fig. 4H). Moreover, the proportion of cells displaying severe chromosomal damage (MCF cells) dropped from 4% to 0.25% (Fig. 4H). These findings suggest that DNA damage correlated with tumors in SHMT-depleted cells was mostly induced by ROS. The increased sensitivity of SHMT-depleted tumor cells to X-ray and HU treatments, coupled with the persistence of γ-H2Av repair foci over time, further suggests that while ROS acts as the primary initiator of DNA damage, the compromised DNA repair and persistent replicative stress due to SHMT depletion amplify the damage downstream, ultimately promoting tumorigenesis (Fig. 5).

### Synergistic interaction between SHMT and pyridoxal 5’-phosphate (PLP) on *Ras*^*V12*^*Dlg*^*RNAi*^ tumor progression

**Fig. 6:**
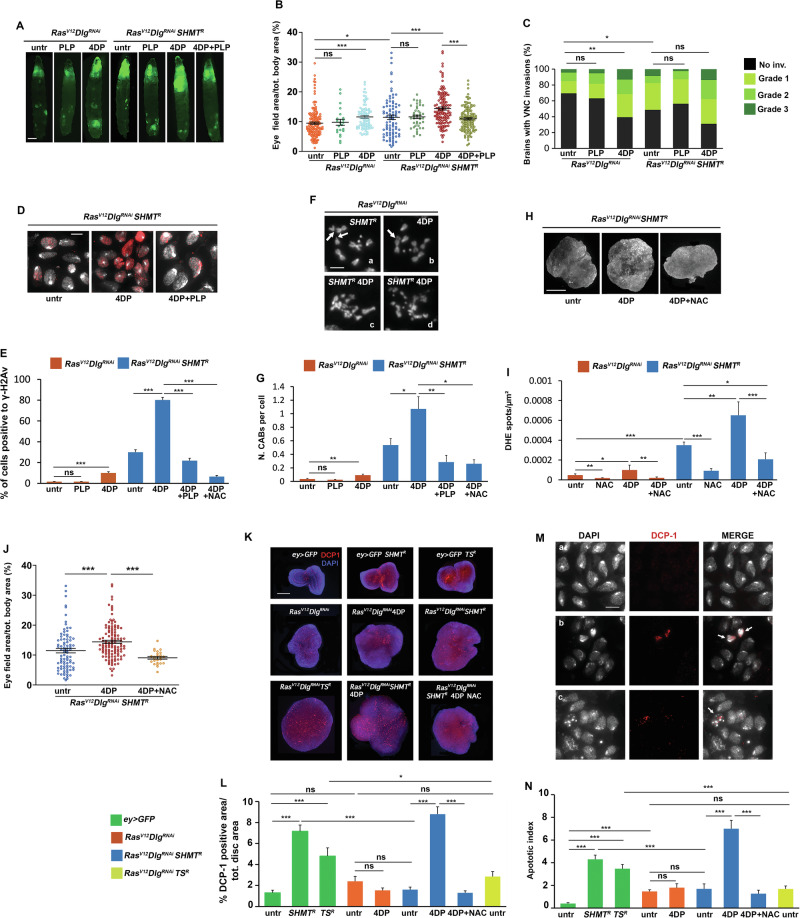
Synergistic interaction between SHMT and PLP on *Ras*^*V12*^*Dlg*^*RNAi*^ tumors. **A** Representative images of larvae showing GFP-labeled tumors. PLP=pyridoxal 5’-phosphate; 4DP = 4-deoxypyridoxine. Scale bar, 0.5 mm. **B** Quantification of GFP-positive eye field area relative to total body area. Error bars, SEM. *P < 0.05; ***P < 0.001 (unpaired *t*-test). Ns=not significant. *Ras*^*V12*^*Dlg*^*RNAi*^ PLP vs *Ras*^*V12*^*Dlg*^*RNAi*^ *P* = 0.78; *Ras*^*V12*^*Dlg*^*RNAi*^*SHMT*^*R*^ PLP vs *Ras*^*V12*^*Dlg*^*RNAi*^*SHMT*^*R*^ *P* = 0.89. Number of larvae scored in at least three independent experiments: *Ras*^*V12*^*Dlg*^*RNAi*^ *n* = 152; *Ras*^*V12*^*Dlg*^*RNAi*^ PLP *n* = 22; *Ras*^*V12*^*Dlg*^*RNAi*^ 4DP *n* = 83; *Ras*^*V12*^*Dlg*^*RNAi*^*SHMT*^*R*^ *n* = 85; *Ras*^*V12*^*Dlg*^*RNAi*^*SHMT*^*R*^ PLP *n* = 41; *Ras*^*V12*^*Dlg*^*RNAi*^*SHMT*^*R*^ 4DP *n* = 119; *Ras*^*V12*^*Dlg*^*RNAi*^*SHMT*^*R*^ 4DP PLP *n* = 123. **C** Quantification of secondary tumors. The green-labeled portion of each column represents the percentage of brains with VNC invasions. The black portion indicates the percentage of brains without invasions. Statistics were assessed by chi square test and refer to the percentage of invasion phenotype *P < 0.05; **P < 0.01. Ns not significant *Ras*^*V12*^*Dlg*^*RNAi*^ PLP vs *Ras*^*V12*^*Dlg*^*RNAi*^ *P* = 0.53; *Ras*^*V12*^*Dlg*^*RNAi*^*SHMT*^*R*^ vs. *Ras*^*V12*^*Dlg*^*RNAi*^*SHMT*^*R*^ PLP *P* = 0.43; *Ras*^*V12*^*Dlg*^*RNAi*^*SHMT*^*R*^ vs *Ras*^*V12*^*Dlg*^*RNAi*^*SHMT*^*R*^ 4DP *P* = 0.09. Number of brains scored in at least three independent experiments: *Ras*^*V12*^*Dlg*^*RNAi*^ *n* = 46; *Ras*^*V12*^*Dlg*^*RNAi*^ PLP *n* = 38; *Ras*^*V12*^*Dlg*^*RNAi*^ 4DP *n* = 38; *Ras*^*V12*^*Dlg*^*RNAi*^*SHMT*^*R*^ *n* = 80; *Ras*^*V12*^*Dlg*^*RNAi*^*SHMT*^*R*^ PLP *n* = 39; *Ras*^*V12*^*Dlg*^*RNAi*^*SHMT*^*R*^ 4DP *n* = 25. **D** Examples of eye disc nuclei showing γ-H2Av foci (red). 4DP treatment strongly increased the percentage of cells positive to γ-H2Av immunostaining. Scale bar, 5 µm. **E** Quantification of results. Error bars, SEM. ***P < 0.001 (unpaired *t*-test). Ns not significant *Ras*^*V12*^*Dlg*^*RNAi*^ PLP vs *Ras*^*V12*^*Dlg*^*RNAi*^ P = 0.85; *Ras*^*V12*^*Dlg*^*RNAi*^*SHMT*^*R*^ 4DP PLP vs *Ras*^*V12*^*Dlg*^*RNAi*^*SHMT*^*R*^ *P* = 0.05 (not reported in the graph). NAC = N-acetyl cysteine (4 mg/ml). Number of scored cells in three independent experiments: *Ras*^*V12*^*Dlg*^*RNAi*^ *n* = 5092; *Ras*^*V12*^*Dlg*^*RNAi*^ PLP *n* = 3500; *Ras*^*V12*^*Dlg*^*RNAi*^ 4DP *n* = 4309; *Ras*^*V12*^*Dlg*^*RNAi*^*SHMT*^*R*^ *n* = 2461; *Ras*^*V12*^*Dlg*^*RNAi*^*SHMT*^*R*^ 4DP *n* = 946; *Ras*^*V12*^*Dlg*^*RNAi*^*SHMT*^*R*^ 4DP PLP *n* = 1514; *Ras*^*V12*^*Dlg*^*RNAi*^*SHMT*^*R*^ 4DP NAC *n* = 2078. **F** Examples of CABs in eye discs of indicated genotypes/treatments. (a) centric deletion of a major autosome (arrows); (b) isochromatid deletion of a major autosome (arrowed); (c,d) metaphases with multiply fragmented chromosome. Scale bar, 5 µm. **G** Quantification of results. Error bars, SEM. *P < 0.05; **P < 0.01 (unpaired *t*-test). Ns = not significant *Ras*^*V12*^*Dlg*^*RNAi*^ PLP vs *Ras*^*V12*^*Dlg*^*RNA*^ *P* = 0.21; *Ras*^*V12*^*Dlg*^*RNAi*^*SHMT*^*R*^ 4DP PLP vs *Ras*^*V12*^*Dlg*^*RNAi*^*SHMT*^*R*^ *P* = 0.08 (not reported in the graph).Total number of examined cells in at least three independent experiments: *Ras*^*V12*^*Dlg*^*RNAi*^ *n* = 538 (8 discs); *Ras*^*V12*^*Dlg*^*RNAi*^ PLP *n* = 294 (3 discs); *Ras*^*V12*^*Dlg*^*RNAi*^ 4DP *n* = 457 (9 discs); *Ras*^*V12*^*Dlg*^*RNAi*^*SHMT*^*R*^ *n* = 501 (16 discs); *Ras*^*V12*^*Dlg*^*RNAi*^*SHMT*^*R*^ 4DP *n* = 197 (4 discs); *Ras*^*V12*^*Dlg*^*RNAi*^*SHMT*^*R*^ 4DP PLP *n* = 443 (6 discs); *Ras*^*V12*^*Dlg*^*RNAi*^ *SHMT*^*R*^ 4DP NAC *n* = 203 (4 discs). **H** ROS accumulation in 4DP-treated *Ras*^*V12*^*Dlg*^*RNAi*^*SHMT*^*R*^ eye discs, detected by dihydroethidium (DHE) staining. NAC treatment (4 mg/ml) reduces ROS increase. Scale bar, 20 μm. **I** Quantification of results. Spot density expressed as the number of DHE-positive spots per square micrometer (μm^2^). Error bars, SEM. *P < 0.05; **P < 0.01; ***P < 0.001 (unpaired *t*-test). Number of scored eye discs in three independent experiments: *Ras*^*V12*^*Dlg*^*RNAi*^*n* = 14; *Ras*^*V12*^*Dlg*^*RNAi*^ NAC *n* = 10; *Ras*^*V12*^*Dlg*^*RNAi*^ 4DP *n* = 13*; Ras*^*V12*^*Dlg*^*RNAi*^ 4DP NAC *n* = 14*; Ras*^*V12*^*Dlg*^*RNAi*^*SHMT*^*R*^ *n* = 16*; Ras*^*V12*^*Dlg*^*RNAi*^*SHMT*^*R*^ NAC *n* = 11; *Ras*^*V12*^*Dlg*^*RNAi*^*SHMT*^*R*^ 4DP *n* = 9; *Ras*^*V12*^*Dlg*^*RNAi*^*SHMT*^*R*^ 4DP NAC *n* = 17. **J** NAC treatment (4 mg/ml) reduces primary tumor area in 4DP-fed *Ras*^*V12*^*Dlg*^*RNAi*^*SHMT*^*R*^ larvae. Quantification of GFP-positive eye field area relative to total body area. Error bars, SEM. P < 0.001 (unpaired *t*-test). Number of scored larvae in three independent experiments: *Ras*^*V12*^*Dlg*^*RNAi*^*SHMT*^*R*^ *n* = 85; *Ras*^*V12*^*Dlg*^*RNAi*^*SHMT*^*R*^ 4DP *n* = 119; *Ras*^*V12*^*Dlg*^*RNAi*^*SHMT*^*R*^ 4DP NAC *n* = 25. **K** 4DP treatment increases apoptosis in *Ras*^*V12*^*Dlg*^*RNAi*^*SHMT*^*R*^ cells. Eye discs of the indicated genotypes and treatments were immunostained with anti-DCP-1 antibody to detect apoptosis. The anti-DCP-1 signal is shown in red. Nuclei were counterstained with DAPI (blue). Scale bar, 20 μm. (NAC, 4 mg/ml) **L** Quantification of results was performed by measuring the percentage area of anti-DCP-1 positive spots relative to the total disc area, analyzed on confocal z-stack maximum intensity projections. Error bars, SEM. *P < 0.051; ***P < 0.001 (unpaired *t*-test). Ns= not significant *Ras*^*V12*^*Dlg*^*RNAi*^ vs *ey* > *GFP* *P* = 0.05; *Ras*^*V12*^*Dlg*^*RNAi*^ 4DP vs *Ras*^*V12*^*Dlg*^*RNA*^ *P* = 0.098; *Ras*^*V12*^*Dlg*^*RNAi*^ *SHMT*^*R*^ vs *Ras*^*V12*^*Dlg*^*RNAi*^ *P* = 0.217; *Ras*^*V12*^*Dlg*^*RNAi*^*TS*^*R*^ vs *Ras*^*V12*^*Dlg*^*RNAi*^ P = 0.22. Number of scored discs in three independent experiments: *ey* > *GFP n* = 22; *ey* > *GFP SHMT*^*R*^ *n* = 15; *ey* > *GFP TS*^*R*^ *n* = 14; *Ras*^*V12*^*Dlg*^*RNAi*^ *n* = 15; *Ras*^*V12*^*Dlg*^*RNAi*^ 4DP *n* = 19; *Ras*^*V12*^*Dlg*^*RNAi*^*SHMT*^*R*^ *n* = 15; *Ras*^*V12*^*Dlg*^*RNAi*^*SHMT*^*R*^ 4DP *n* = 21. *Ras*^*V12*^*Dlg*^*RNAi*^*SHMT*^*R*^4DP NAC *n* = 12; *Ras*^*V12*^*Dlg*^*RNAi*^*TS*^*R*^ *n* = 15. **M** Examples of apoptosis detection by anti-DCP-1 immunofluorescence on squashed eye disc preparations. (a) Cells showing normal nuclear morphology and no activation of DCP-1; (b) Nuclei displaying strong chromatin condensation with intense anti-DCP-1 staining (arrows); (**c**) Nucleus exhibiting chromatin fragmentation, and anti-DCP-1 signal. Scale bar, 5 μm. **N** Quantification. Apoptotic Index (%) = DCP-1 positive cells number/total number of examined cells. Error bars, SEM. ***P < 0.001 (unpaired *t*-test). Ns= not significant. *Ras*^*V12*^*Dlg*^*RNAi*^ 4DP vs *Ras*^*V12*^*Dlg*^*RNAi*^ *P* = 0.52; *Ras*^*V12*^*Dlg*^*RNAi*^*SHMT*^*R*^ vs *Ras*^*V12*^*Dlg*^*RNAi*^ *P* = 0.27; *Ras*^*V12*^*Dlg*^*RNAi*^*TS*^*R*^ vs *Ras*^*V12*^*Dlg*^*RNAi*^ *P* = 0.27. Number of scored cells*: ey* > *GFP n* = 7430; *ey* > *GFP SHMT*^*R*^ *n* = 3452; *ey* > *GFP TS*^*R*^ *n* = 3722; *Ras*^*V12*^*Dlg*^*RNAi*^ *n* = 3612; *Ras*^*V12*^*Dlg*^*RNAi*^4DP *n* = 4612; *Ras*^*V12*^*Dlg*^*RNAi*^*SHMT*^*R*^ *n* = 2414; *Ras*^*V12*^*Dlg*^*RNAi*^*SHMT*^*R*^ 4DP *n* = 3077; *Ras*^*V12*^*Dlg*^*RNAi*^*SHMT*^*R*^ 4DP NAC *n* = 2595; *Ras*^*V12*^*Dlg*^*RNAi*^*TS*^*R*^ *n* = 2539.

SHMT uses the catalytically active form of vitamin B6, the pyridoxal 5’-phosphate (PLP), as a cofactor to perform the reversible conversion of serine and THF to glycine and 5,10-methylene THF (Fig. 1A). In addition, PLP is an antioxidant molecule whose depletion causes DNA damage [44]. We previously demonstrated an interaction between SHMT and PLP which affects chromosome damage [28], leading us to hypothesize that this interaction could also influence cancer progression. Consequently, we examined whether a reduction in PLP levels, achieved by feeding larvae with the PLP antagonist 4-deoxypyridoxine (4DP) [45] influenced *Ras*^*V12*^*Dlg*^*RNAi*^ *SHMT*^*RNAi*^ tumor progression. 4DP-fed *Ras*^*V12*^*Dlg*^*RNAi*^*SHMT*^*RNAi*^ larvae displayed a primary tumor area covering about 14% of the larval body, compared to 11.4% in untreated *Ras*^*V12*^*Dlg*^*RNAi*^ *SHMT*^*RNAi*^ controls (Fig. 6A, B). Regarding tumor invasiveness, VNC invasion was observed in 69% of the examined brains from 4DP-fed *Ras*^*V12*^*Dlg*^*RNAi*^*SHMT*^*RNAi*^ larvae (Fig. 6C); however, this latter value was not statistically significant when compared to either *Ras*^*V12*^*Dlg*^*RNAi*^*SHMT*^*RNAi*^ (51%) or 4DP-fed *Ras*^*V12*^*Dlg*^*RNAi*^ (60.5%).

PLP supplementation to 4DP-fed *Ras*^*V12*^*Dlg*^*RNAi*^*SHMT*^*RNAi*^ larvae decreased the size of the GFP-labeled cephalic area (10.95%) (Fig. 6A, B), thus suggesting the specificity of 4DP treatment.

Interestingly, 4DP treatment in *Ras*^*V12*^*Dlg*^*RNAi*^*SHMT*^*RNAi*^ larvae strongly increased the percentage of γ-H2Av foci-positive cells to 80%, highlighting a synergistic effect between SHMT depletion and reduced cofactor availability. This 80% incidence was, indeed, greater than the sum of the individual effects of *Ras*^*V12*^*Dlg*^*RNAi*^*SHMT*^*RNAi*^ (31.4%) and *Ras*^*V12*^*Dlg*^*RNAi*^4DP (10%) conditions (*P* < *0.001*). PLP supplementation markedly reduced this effect (21%), a reduction that was not statistically significant compared to *Ras*^*V12*^*Dlg*^*RNAi*^*SHMT*^*RNAi*^ cells (Fig. 6D, E).

A synergistic effect was also observed for chromosome damage. Eye discs from 4DP-fed *Ras*^*V12*^*Dlg*^*RNAi*^*SHMT*^*RNAi*^ larvae exibited a frequency of 1.07 CABs per cell (Fig. 6F, G). This value exceeded the cumulative sum observed in *Ras*^*V12*^*Dlg*^*RNAi*^*SHMT*^*RNAi*^ (0.54) and in 4DP-fed *Ras*^*V12*^*Dlg*^*RNAi*^ (0.091) larvae, thereby confirming a synergistic effect for PLP reduction and SHMT depletion (*P* < *0.01*) in promoting genomic instability. Also in this case, PLP administration to 4DP-fed *Ras*^*V12*^*Dlg*^*RNAi*^*SHMT*^*RNAi*^ larvae rescued 4DP-induced increase in chromosome aberrations, restoring CAB levels closer to baseline (Fig. 6F, G).

4DP treatment further increased ROS accumulation in *Ras*^*V12*^*Dlg*^*RNAi*^*SHMT*^*RNAi*^ eye discs, a finding also in agreement with the role of PLP in oxidative stress [44] (Fig. 6H, I). Consistently, the administration of 4 mg/ml NAC to 4DP-fed *Ras*^*V12*^*Dlg*^*RNAi*^*SHMT*^*RNAi*^ larvae reduced ROS (Fig. 5H, I), γ-H2Av foci accumulation (Fig. 6D, E), CAB frequency (Fig. 6F, G) and primary tumors (Fig. 6J). In contrast, the concentration of 1 mg/ml NAC used in the aforementioned experiments did not induce a statistically significant rescue, possibly due to the huge oxidative stress.

To understand why the combined depletion of SHMT and PLP leads to a synergistic increase in DNA damage but not a corresponding enhancement in tumor progression, we examined apoptosis levels. Apoptosis was evaluated using an antibody against Death Caspase-1 (DCP-1), a critical effector of the process. Immunofluorescence experiments were performed on both whole mount eye discs and squashed preparations to better evaluate the number of apoptotic cells (Fig. 6K–N). This analysis revealed high levels of cell death in *Ras*^*V12*^*Dlg*^*RNAi*^*SHMT*^*RNAi*^ cells treated with 4DP (Fig. 6K–N). Interestingly, while 4.3% of cells were positive for DCP-1 in *ey* > *GFP SHMT*^*RNAi*^ non-tumor cells, the *Ras*^*V12*^*Dlg*^*RNA*^*SHMT*^*RNAi*^ eye discs displayed only 1.7% of positive cells, Fig. 6N). This indicates that the *Ras*^*V12*^*Dlg*^*RNAi*^ background appears to exert an anti-apoptotic effect, permitting the survival of SHMT (or TS) depleted cells, despite their more severe chromosomal damage. In contrast, 4DP treatment effectively bypassed this intrinsic resistance to cell death, restoring apoptosis even within the tumor context (7% of apoptotic cells). Similar results were obtained by Acridine orange (AO) staining (Figure S8).

## Discussion

Our work explores, for the first time in vivo, the tumor suppressor role of SHMT and identifies DNA damage as a key underlying mechanism. Moreover, we show that PLP depletion in SHMT-deficient cancer cells can influence cancer progression.

*Drosophila* is an ideal cancer model, due to its conserved metabolic and oncogenic pathways and robust genetic tools [46]. A key advantage is the presence of a single *SHMT* gene, eliminating concerns about genetic redundancy. The *Ras*^*V12*^*Dlg*^*RNAi*^ cancer model was particularly suitable for this study as it maintains normal SHMT expression levels.

*Ras*^*V12*^*Dlg*^*RNAi*^ cancers receive mitogenic signals from the conserved JAK-STAT and MAPK pathways and affect the epithelial tissue [47], mirroring human carcinomas, which represent up to 90% of human cancers.

Our analysis demonstrates that *SHMT* silencing enhances the progression of *Ras*^*V12*^*Dlg*^*RNAi*^ tumors by impairing folate pathway (Fig. 1A), specifically dTMP biosynthesis. Silencing the downstream enzyme TS (which produces dTMP from dUMP) phenocopies SHMT silencing. Notably, dTMP administration rescues *Ras*^*V12*^*Dlg*^*RNAi*^*SHMT*^*RNAi*^ tumor progression, suggesting that the folate pathway is functionally dominant over the methionine cycle here. This rescue also implies that low SHMT levels may preferentially redirect methylene tetrahydrofolate (me-THF) towards the methionine cycle. Since SHMT1 in human cells channels me-THF toward nuclear dTMP synthesis by sequestering 5-methylTHF (5-mTHF) in the cytoplasm [21, 48], we hypothesize that low SHMT levels reduce m-THF sequestration, thus favoring the methionine cycle and further reducing dTMP biosynthesis.

A critical finding is that ROS-induced DNA damage triggered by low dTMP levels drives tumor progression in *Ras*^*V12*^*Dlg*^*RNAi*^*SHMT*^*RNAi*^ cells. These cells exhibited high levels of ROS, DSBs, and CABs. Consistent with the SHMT role in purine and pyrimidine biosynthesis, *Ras*^*V12*^*Dlg*^*RNAi*^*SHMT*^*RNAi*^ cells resulted highly sensitive to genotoxic stressors such as HU and X-rays. They also displayed defective DNA repair (evidenced by impaired resolution of X-ray-induced γ-H2Av repair foci). However, although replicative stress and impaired repair are known contributors to genome instability, our data identify ROS as the principal driving factor of cancer progression. Treatment with the antioxidant NAC reduced ROS and rescued tumor progression and DNA damage. This effect mirrors the dTMP treatment, which resolves both replication/repair issues and oxidative stress. Thus, we propose a primary pathway wherein SHMT depletion leads to increased ROS, consequently inducing DSBs that result in CABs and tumorigenesis (Fig. 5). Secondary mechanisms—including replicative stress and impaired repair—likely contribute to a lesser extent to overall genomic instability. Specifically, replicative stress may primarily drive DSB formation [49] whereas defective repair may further facilitate the transformation of DSBs into CABs [37].

Although ROS increase can impact tumorigenesis through diverse mechanisms including altered signaling pathways, chronic inflammation, and microenvironmental modulation [50], the concomitant rescue of both DNA damage and tumor phenotypes achieved with the antioxidant NAC strongly suggests that, in our model, ROS act primarily via DNA damage. Furthermore, these results strongly suggest that genome instability plays a crucial role in cancer progression. Although dUTP incorporation (caused by SHMT depletion) is a potential source of DNA damage, the rescue effect of NAC suggests that this mechanism is not relevant in our cancer model. In line with our findings, the downregulation of SHMT in human hepatocellular carcinoma [19] also results in elevated ROS levels. However, in that specific context, the oxidative stress promotes cancer progression by altering the expression of genes involved in epithelial-mesenchymal transition and spreading.

Although NAC or dTMP treatments fully rescued tumor phenotypes, they left some residual chromosome damage. However, by segmenting the data based on the extent of chromosomal damage, we observed that NAC or dTMP treatments more effectively rescued the class of cells showing more severe chromosome damage (MCF cells) than the less damaged class. This suggests that a low level of genomic instability might be tolerable and insufficient to drive severe tumorigenesis, raising the hypothesis of a critical threshold.

Our model assigns a central role to dTMP depletion. The successful rescue of ROS accumulation through dTMP supplementation strongly implies that dTMP depletion drives ROS production; however, the precise underlying mechanism was not clarified in this study. While NADPH oxidase upregulation has been proposed as the main driver for ROS increase in dTMP-depleted cells [19, 40], we found that *Ras*^*V12*^*Dlg*^*RNAi*^*SHMT*^*RNAi*^ cells exhibited reduced levels of this enzyme (Figure S9), and consistently the NOX inhibitor VAS2879 failed to rescue tumor progression, pointing toward the involvement of a NOX-independent mechanism. Although chromosome damage was not assessed in secondary tumors, genomic instability in primary lesions is a known contributor to metastatic spread [51]. The reduction in brain invasions observed after dTMP or NAC treatments (which mitigated DNA damage) suggests that DNA damage can influence secondary tumor formation. However, we cannot exclude the contribution of other ROS-mediated mechanisms.

While more than 30% of all human cancers are driven by mutations of the RAS family of genes [52, 53] and the loss of cell polarity induced by mammalian orthologs of DLGs (DLG1-5) characterizes many human tumors [54–56], the specific co-occurrence of RAS hyperactivation and DLG depletion is uncommon in human tumors likely due to redundancies among DLG paralogs. Nevertheless, the *Drosophila Ras*^*V12*^*Dlg*^*RNAi*^ system represents an invaluable platform for identifying tumor modifiers [31–33] —such as SHMT—whose mechanistic role is likely applicable and transferable across a broad range of human malignancies. Consistently, we found that reduced SHMT activity also affected the *Ras*^*V12*^*csk*^*-/-*^ tumor model, a system sensitive to metabolic alterations [36].

We also found that intracellular PLP levels can significantly modulate the impact of SHMT depletion on *Ras*^*V12*^*Dlg*^*RNAi*^*SHMT*^*RNAi*^ cancers. Crucially, our study provides the first in vivo evidence of a SHMT-PLP gene-nutrient interaction influencing cancer progression through DNA damage. We observed that the concomitant depletion of SHMT and its cofactor PLP led to only a modest increase in primary cephalic tumor growth compared to SHMT depletion alone. In contrast, a pronounced synergistic effect between SHMT and PLP depletions was observed affecting DNA and chromosome damage. Thus, we propose that in *Ras*^*V12*^*Dlg*^*RNAi*^*SHMT*^*RNAi*^ cells, 4DP treatment may induce such an extensive DNA damage that it drives the cells towards apoptosis, as confirmed by observed elevated apoptosis rate, thereby limiting excessive cell proliferation. The basis of this synergism can be an intensified oxidative stress due to both a further reduction of SHMT activity induced by PLP depletion and a weakening of PLP antioxidant function. This is further confirmed by elevated ROS levels in *Ras*^*V12*^*Dlg*^*RNAi*^*SHMT*^*RNAi*^ cells treated with 4DP, and the observed rescue induced by NAC administration. In accordance with this hypothesis previous findings in *Drosophila* as well as in mammals [28, 57] indicate that low PLP levels can reduce SHMT activity. Furthermore, antioxidant treatments such as ascorbic acid or α-lipoic acid as well as catalase overexpression, were able to rescue both tumor growth and CABs in *Ras*^*V12*^*Dlg*^*RNAi*^ larvae reared on 4DP-supplemented medium [33].

Translated to human cancers our findings may suggest that in tumors with reduced SHMT activity, PLP depletion worsens the outcome by exacerbating oxidative stress, though this effect may be partially mitigated by apoptosis. Conversely, in tumors that overexpress SHMT, a therapeutic strategy involving SHMT inhibitors along with either PLP inhibitors or the silencing of genes involved in PLP biosynthesis might be a promising way to inhibit cancer progression. A recent study revealed that the anti-diabetic metformin [35] can disrupt the PLP-dependent SHMT2 oligomerization, thus decreasing SHMT2 activity in cancer cells that overexpress the enzyme. Our data further suggest that depleting PLP could increase oxidative stress in SHMT-overexpressing tumors, simultaneously impairing its cofactor function and its role as an antioxidant molecule. However, given the essential roles of PLP, a targeted approach is crucial to prevent systemic toxicity.

In conclusion, our comprehensive investigation establishes SHMT role as a tumor suppressor and demonstrates that its depletion drives tumor formation through DNA damage primarily mediated by ROS, exacerbated by impaired DNA repair and replication stress. Importantly, we have identified a significant gene-nutrient interaction between SHMT and vitamin B6, directly impacting these same tumor phenotypes with potential therapeutic implications.

## Materials and methods

### *Drosophila* stocks and crosses

*SHMT*^*v19206*^ and *TS*^*v29354*^ lines were obtained from Vienna *Drosophila* Resource Center (VDRC).

*eyflp;UAS*-*Ras*^*V12*^, *UAS*-*Dlg*^*RNAi*^/*CyO*, *Gal80*; *act* **>** *CD2*>*Gal4*, *UAS*-*GFP* and *eyflp*; *Sp*/*CyO*, *Gal80*; *act* **>** *CD2*>*Gal4*, *UAS*-*GFP* stocks were obtained by K. Basler (Institute of Molecular Life Sciences, University of Zurich, Switzerland). *UAS*-*Ras*^*V12*^/*UAS*-*Ras*^*V12*^; *FRT82B*/*FRT82B* and *UAS*-*Ras*^*V12*^; *FRT82B* *csk*^*Q156*^/*TM6B* were kindly provided by Hirabayashi lab (London, Institute of Medical Science). *yw eyFlp*; *Act* **>** *y* **+** >*Gal4* *UAS*-*GFP*; *FRT82B*, *Tub Gal80* was kindly provided by T. Xu lab (Yale School of Medicine).

All stocks were maintained at 25°C. The used balancers and genetic markers are described in detail in FlyBase (http://flybase.bio.indiana.edu/).

### Genetic crosses

-To generate larvae carrying *Ras*^*V12*^ *Dlg*^*RNAi*^ tumors, *eyflp; UAS-Ras*^*V12*^*, UAS-Dlg*^*RNAi*^*/CyO, Gal80; act* > *CD2>Gal4, UAS-GFP* females were mated to *Oregon-R* males.

-To generate *Ras*^*V12*^*Dlg*^*RNAi*^ larvae depleted of SHMT or TS, *eyflp;UAS-Ras*^*V12*^*, UAS-Dlg* ^*RNAi*^*/CyO, Gal80; act* > *CD2>Gal4, UAS-GFP* females were mated to *SHMT or TS RNAi lines*.

-Control larvae expressing only the GFP protein (depleted or not for *SHMR* or *TS*) in the eye-antennal discs were obtained by crossing: *eyflp; Sp/CyO,Gal80; act* > *CD2>Gal4, UAS-GFP* females to *Oregon-R* males (or *SHMT* or *TS* RNAi lines).

-To generate larvae expressing *Ras*^*V12*^ in eye discs *eyflp; Sp/CyO,Gal80; act* > *CD2>Gal4, UAS-GFP* females were crossed to *UAS-Ras*^*V12*^*/ UAS-Ras*^*V12*^ males.

-To generate *Ras*^*V12*^*csk*^*-/-*^ larvae *yw eyFlp; act* > *y* + *> Gal4 UAS GFP; FRT82B, Tub Gal80* females were crossed to *UAS-Ras*^*V12*^*; FRT82B csk*^*Q156*^*/TM6B* males.

### Treatments

All stocks were maintained and crossed at 25 °C on a standard medium containing: 0.68 g. agar, 6.52 g. yeast, 3 g. flour, 600 µL propionic acid, and 5.13 g. sucrose, in 100 mL.

4-deoxypyridoxine (4DP, Sigma Cat. No. D0501) as well as pyridoxal 5’phosphate (PLP, Sigma Cat. No. P9255), were dissolved in the standard medium at 2 mM and 0.5 mM final concentrations, respectively. These concentrations were chosen according to [44].

Deoxythymidine monophosphate (dTMP, Merck Cat. No. T7004-100MG) was dissolved in the standard medium at 200 μM concentration according to [28, 33].

N-acetyl cysteine (Sigma, Cat. No. A9165) was dissolved in the standard medium at 1 mg/ml or 4 mg/ml according to [58].

1,1-Dimethylbiguanide hydrochloride (Metformin) Sigma, Cat. No D150959 was dissolved in the standard medium at 50 mM concentration according to [59].

### Analysis of larvae

*Ras*^*V12*^*Dlg*^*RNAi*^ third instar larvae (carrying or not carrying *SHMT* or *TS* interfering constructs) were collected at 12–14 days after the cross due to a developmental delay. Conversely, non-tumor *ey* > *GFP* larvae (carrying or not carrying *SHMT* or *TS* interfering constructs) were collected at 6–8 days after the cross. For primary tumor analysis, larvae were immobilized in PBS at 4 °C for at least 30 minutes and then examined using Nikon Eclipse E600 fluorescence microscope, equipped with a mercury lamp and a CDD camera (CoolSNAP MYO). Measurements of the GFP-labeled area were performed on the acquired images using ImageJ 1.54 g software (details are provided in Figure S2). Larvae from at least 3 independent experiments have been examined.

### Quantification of VNC invasions

Brains from wandering third-instar larvae collected were dissected in saline (NaCl 0.7%) and examined at the fluorescence microscope (Nikon). The invasions of GFP clones from their original sites (eye-antennal discs and optical lobes) to ventral nerve cords (VNCs) were considered as secondary tumors.

### Chromosome cytology

To analyze chromosome aberrations (CABs), eye discs from third instar larvae were dissected in saline (NaCl 0.7%) and incubated in colchicine (final concentration 10^-5 ^M) for 55 minutes. The eye discs were then incubated in hypotonic solution (sodium citrate 0.5%) for 9 minutes, squashed in 45% acetic acid and frozen in liquid nitrogen.

Preparations were mounted in Vectashield H-1200 with 4,6-diamidino-2-phenylindole (DAPI) (Vector Laboratories, Burlingame, CA) to stain the DNA. Cytological preparations were examined with a Nikon Eclipse E600 fluorescence microscope, equipped with a mercury lamp and a CDD camera (CoolSNAP MYO). The number of total cells scored, and the number of scored eye discs are reported in the figure legends. To calculate the number of CABs per cell, we arbitrarily assigned five CABs to each cell with multifragmented chromosomes.

### Immunofluorescence

Immunostaining on squashed eye discs from third instar larvae was performed as described in [33]. Eye disc preparations were incubated overnight at 4 °C with primary antibody diluted in PBT (Phosphate Buffered Saline + 0.3% Triton X-100). The primary antibodies used were rabbit anti-Histone H2AvD pS137 Rockland Cat. No. 600-401-914 at 1:100 dilution and rabbit anti-DCP-1 (Cell Signaling Technology Cat. No. 9578) at 1:100 dilution. Following two rinses in PBT primary antibodies were detected by incubation for 1 h with the Alexa Fluor 555-conjugated anti-rabbit secondary antibody (Thermo Fisher Scientific Cat. No. A31572), which was diluted 1:300 in PBT. Observations were carried out using a Nikon Eclipse E600 fluorescence microscope, equipped with a mercury lamp and a CDD camera (CoolSNAP MYO). The γ-H2AV or anti-DCP-1 positive cells were quantified on the acquired pictures using Adobe Photoshop 26.8.1.

Whole mount Immunofluorescence (IF) on eye discs using the antibody anti-DCP-1 (Cell Signaling Technology Cat. No. 9578, 1:100) was performed as described in [33]. Secondary antibody, Alexa Fluor 555-conjugated anti-rabbit (Thermo Fisher Scientific Cat. No. A31572), was diluted 1:500.

Imaging was performed by Zeiss AXIO Observer Z1 inverted fluorescence microscope equipped with an Axiocam 702 mono camera. For each imaginal disc, a minimum of ten slices were acquired. Apoptosis was quantified by determining the percentage area of the anti-DCP-1 positive spots relative to the total disc area, analyzed on confocal z-stack projection images.

### X-ray sensitivity test

Third instar larvae were irradiated with 2.5 Gy of X-rays using the MHF200D Gilardoni (Italy) machine, equipped with an X-ray tube. About three hours later eye antennal discs were dissected and incubated for 55 min in colchicine (final concentration 10^−5 ^M) and then fixed as described above to perform chromosome analysis.

To follow the kinetics of γ-H2Av foci, third instar larvae were irradiated with 5 Gy of X-rays; larval eye discs were then dissected and fixed at various post irradiation (PIR) times as reported in the test.

### HU sensitivity test

Eye antennal discs, dissected from third instar larvae, were incubated for 20 min in saline with 1 mM (or 2 mM in Figure S7) HU (Sigma, Cat. No. H-8627), washed and placed in saline for 2 h 30 min. 55 min before fixation, HU-treated discs were incubated in 10^−5 ^M colchicine. Following hypotonic treatment (9 min in 0.5% sodium citrate), HU-treated discs were fixed as described above to perform chromosome analysis.

### Dihydroethidium (DHE) staining

To assess ROS accumulation, eye-antennal discs from third instar larvae were dissected in Schneider’s medium (Gibco, Cat. No. 21720024) and incubated for 5 min in a dark chamber at room temperature with 30 µM Dihydroethidium (DHE) (Thermo Fisher Cat. No. D23107) in Schneiders medium. Oxidation of DHE by superoxide radicals yields 2-hydroxyethidium. This compound subsequently intercalates into DNA, generating a quantifiable signal at 550 nm within cells characterized by ROS production [60]. After two washes in Schneiders medium, one wash in 0.7% formaldehyde and one wash in PBS, discs were immediately mounted in Fluoromount Mounting Medium (Sigma, Cat. No F4680). Images were captured using a fluorescence microscope (Nikon Eclipse E600), equipped with a mercury lamp and a CDD camera (CoolSNAP MYO).

Quantification was performed on the acquired pictures by using ImageJ software and was expressed as average spot density (number of DHE-positive spots per square micrometer).

### Acridine orange staining

Eye imaginal discs from third instar larvae were dissected in 1xPBS and incubated in 1μg/ml Acridine orange (Sigma, Cat. No. 318337) in PBS for 15 min in a dark chamber. After 2 washes in 1x PBS for 5 min eye discs were mounted on microscope slides with 1x PBS. The images were immediately captured using a fluorescence microscope (Nikon Eclipse E600), equipped with a mercury lamp and a CDD camera (CoolSNAP MYO). Quantification was performed on the acquired pictures by using ImageJ/Fiji software and expressed average spot density (number of AO-positive spots per square micrometer).

### RNA Extraction, Reverse Transcription, and RT-qPCR

RNA was extracted using the NucleoSpin RNA kit (Macherey and Nagel, Bethlehem, PA, USA) from three biological replicates, each made of 100 larval eye discs. RNA concentration and quality were evaluated by measuring in 0.1 N NaOH the OD at 260 nm and the ratio at 260/280 nm, respectively, and by electrophoresis on 1.2% agarose gels. Reverse transcription of DNase-treated RNAs (1 μg) was carried out using the OneScript® Plus cDNA Synthesis Kit (ABM Good, Richmond, BC, Canada) with the random primers provided in the kit. RT-qPCR was performed on a CFX Connect Real Time PCR system (Bio-Rad, Hercules, CA, USA) with a two-step reaction using SYBR green ExcelTaq™ Master Mix (SMOBIO, Hsinchu City, Taiwan) and the oligonucleotides reported below. The relative expression of each target gene was determined by the Pfaffl method using the α-tubulin and EF-1 as normalizers. The fold induction resulting from the different pairs of samples was averaged and the p value was calculated using the Student’s t-test.

### Primers

*SHMT*

for: 5’-CAGCCTTATTCCGGATCCCC-3’

rev: 5’-AATCGATGATGCCCGTCTCC-3’.

*TS*:

for: 5’-CAGCCTTATTCCGGATCCCC-3’

rev: 5’-AATCGATGATGCCCGTCTCC-3’.

*NOX*:

for: 5’-TTTTAACTTCCGTCCCGGCG-3’

rev: 5’-CTGCTCCCGCTCAAAGTAGC-3’.

*tubulin*:

for: 5’-TGTCGCGTGTGAAACACTTC-3’

rev: 5’-AGCAGGCGTTTCCAATCTG-3’.

*EF-1*:

for: 5’-GCGTGGGTTTGTGATCAGTT-3’

rev: 5’-GATCTTCTCCTTGCCCATCC-3’.

### SHMT activity measurement

SHMT catalytic activity was determined as described in [33]. The analysis was performed on two biological replicates per condition with each replicate consisting of 200 eye imaginal discs.

### Statistical analysis

All data are expressed as mean ± standard error of the mean (SEM) from at least three independent experiments. Statistical significance was performed using the unpaired two-tailed *t-*test, Chi square test or Chi square test for independence as indicated in each figure legend. *P* < 0.05 was considered significant. Statistical parameters of individual experiments (value of n, mean, SEM, P values) are reported in each figure legend.

## Supplementary information

Supplementary figures

## Data Availability

All data reported in this paper will be shared upon request.
