# Correction: Tumor suppressor function of SHMT in a *Drosophila Ras*^*V12*^*Dlg*^*RNAi*^ model: DNA damage and synergistic gene-nutrient interaction with PLP

**DOI:** 10.1038/s41419-026-08996-4

**Published:** 2026-07-06

**Authors:** Chiara Angioli, Angelo Ferriero, Eleonora Pilesi, Giulia Tesoriere, Beatrice Agostini, Angela Tramonti, Roberto Contestabile, Fiammetta Vernì

**Affiliations:** 1 Dept. of Biology and Biotechnology “Charles Darwin”, Sapienza University of Rome, Rome, Italy; 2 Institute of Molecular Biology and Pathology, National Research Council, Rome, Italy; 3 Department of Biochemical Sciences “A. Rossi Fanelli”, Sapienza, University of Rome, Rome, Italy; 4 Istituto Pasteur-Fondazione Cenci Bolognetti, Sapienza, University of Rome, Rome, Italy

**Keywords:** Cancer, Diseases

Correction to: *Cell Death & Disease* 10.1038/s41419-026-08602-7, published online 26 March 2026

In Figure 6K, the label “4DP” should be added to the lower central panel corresponding to RasV12DlgRNAi SHMTRNAi, as it identifies the treatment applied to the depicted disc. The omission affects the clarity and correct interpretation of the experimental condition shown in the figure. The error likely occurred during figure preparation due to a Photoshop layer issue.

Incorrect figure
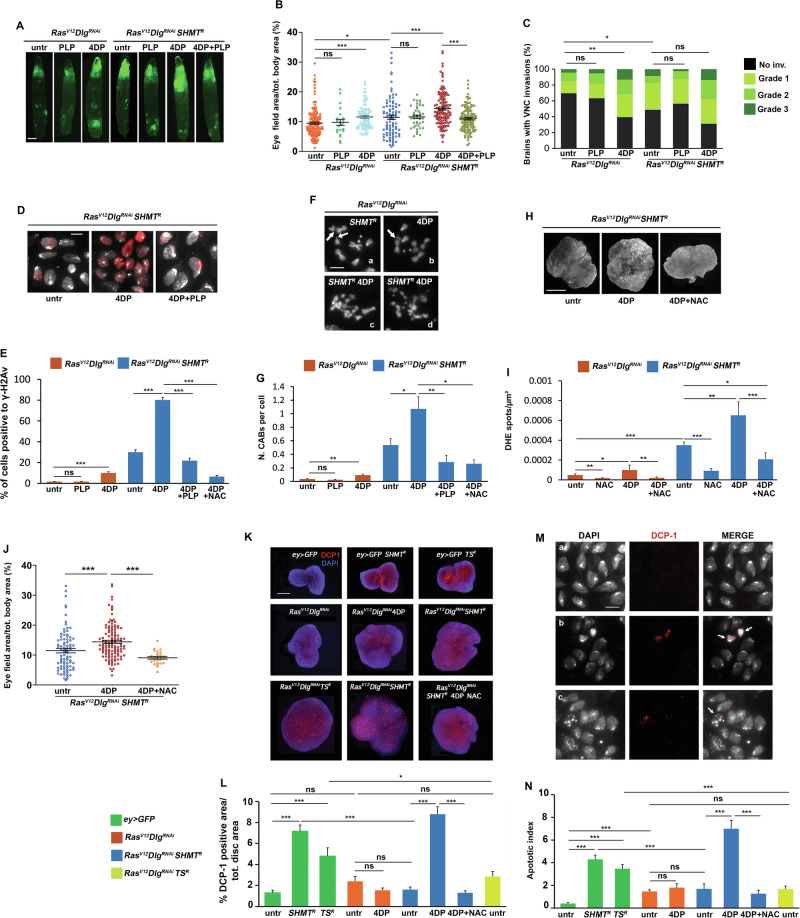


Correct figure
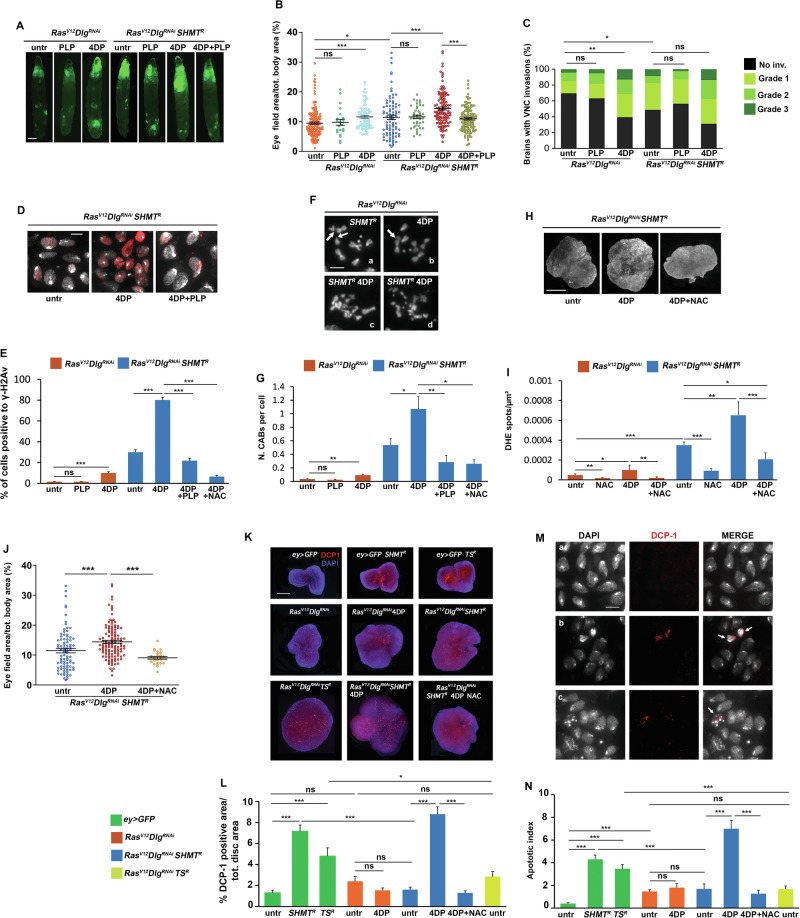


The original article has been corrected.

