# Supplementary material for: Tumor suppressor function of SHMT in a Drosophila RasV12DlgRNAi model: DNA damage and synergistic gene-nutrient interaction with PLP
Source: Cell Death Dis. 2026 Mar 26;17(1):427. doi: 10.1038/s41419-026-08602-7 (PMC13153359; doi:10.1038/s41419-026-08602-7)
Supplement: Supplementary file 1 — Supplementary figures [file 41419_2026_8602_MOESM1_ESM.docx]

**
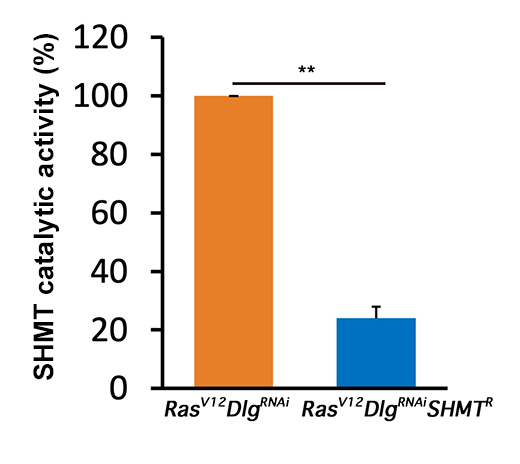
**

**Figure S1** RNA interference significantly reduces the SHMT catalytic activity in eye discs. Error bars, SEM. **P<0.01 (unpaired *t*-test).

**
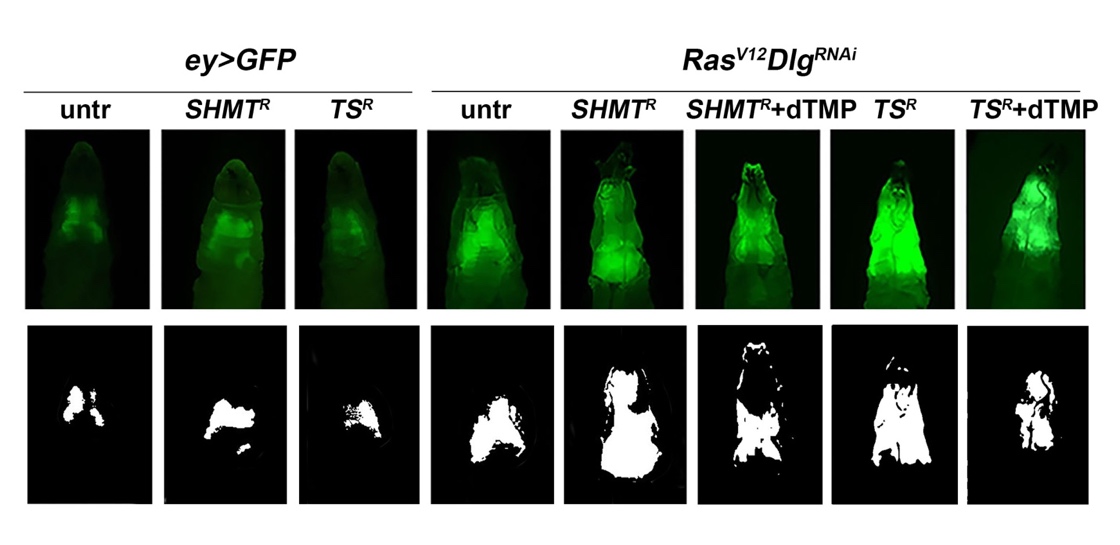
**

**Figure S2** Tumor size measurement in cephalic areas. Quantification of the GFP-positive tumor area was performed using a semi-automated thresholding-based image analysis in ImageJ software. For each larva exemplified in the upper panel (taken from Fig. 1C), a threshold value was set to segment the pixels emitting GFP fluorescence (the tumor) from the background. Although automatic algorithms were used to objectively determine the threshold, a manual check was always performed. Once the GFP-positive pixels were segmented, the total area was calculated (lower panel) by the software. The total area of the larval body was also manually selected and measured. The final percentage of tumor area was calculated as the ratio of the GFP-positive cephalic area to the total area of the larval body.


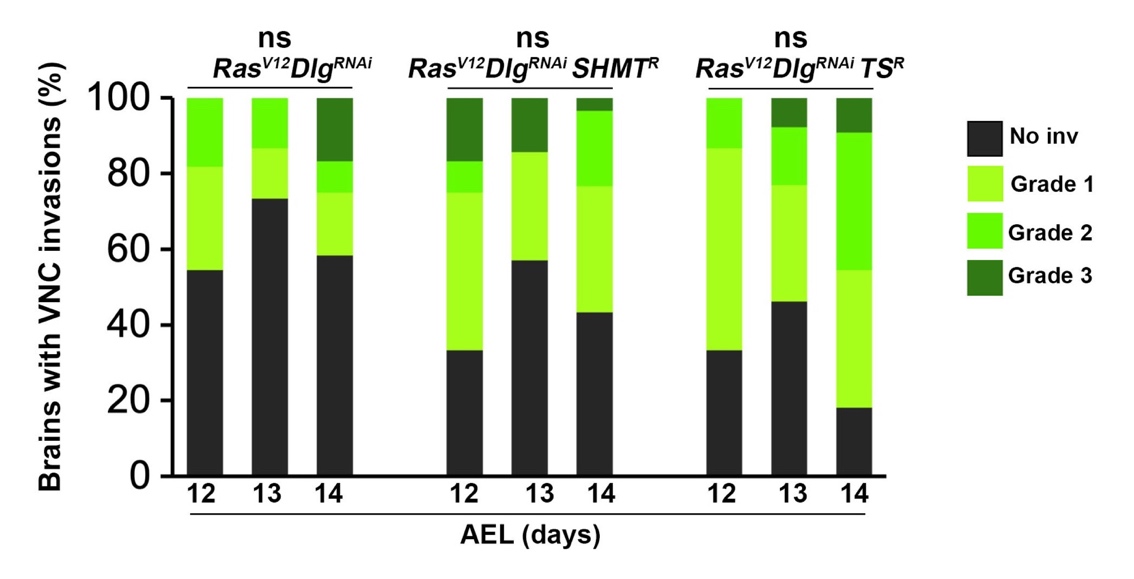


**Figure S3** Correlation between tumor grade and larval age. Grade of tumor invasiveness on the ventral nerve cord (VNC) was quantified for individual larvae across different developmental stages (12 to 14 days after egg laying, AEL). Synchronous larval cohorts were established by restricting egg deposition to a 4-hour window. Statistical analysis (Chi square test for independence) revealed no significant difference in the invasiveness grade among the tested larval ages, indicating that VNC invasion is age-independent within this time window. Ns=not significant *Ras^V12^Dlg^RNA^*^i^ P=0.43; *Ras^V12^Dlg^RNA^SHMT^R^* P=0.21; *Ras^V12^Dlg^RNA^TS^R^* P=0.48. Number of examined larvae: *Ras^V12^Dlg^RNAi^* *n=*40; *Ras^V12^Dlg^RNAi^SHMT^R^ n=*45; *Ras^V12^Dlg^RNAi^TS^R^* *n=*42.

**
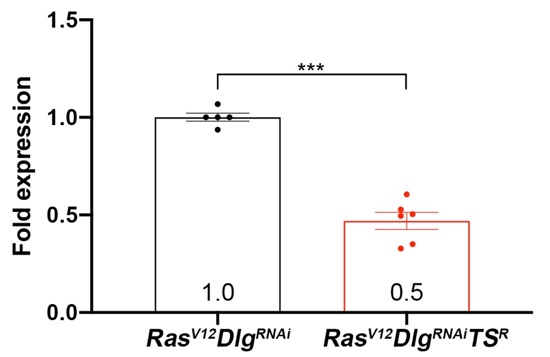
**

**Figure S4** mRNA *TS* levels evaluated by RT-qPCR analysis. The RNAi-mediated silencing of *TS* significantly decreases its expression levels. Error bars, SEM. ***P<0.001 (unpaired *t*-test). *TS^R^=TS^RNAi^*


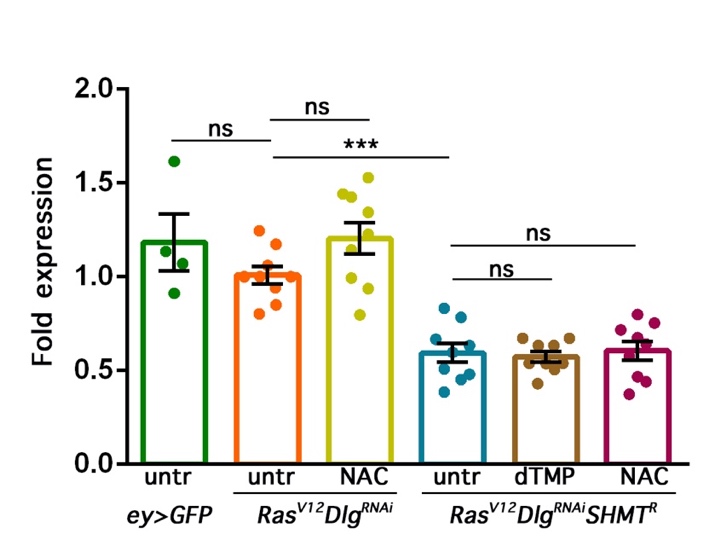


**Figure S5** dTMP or NAC treatments do not alter *SHMT* expression levels. Expression levels of *SHMT* in the indicated genotypes/treatments evaluated by RT-qPCR analysis. Error bars, SEM. ***P<0.001 (unpaired *t*-test). Ns=not significant *ey>GFP* vs *Ras^V12^Dlg^RNAi^* P=0.17; *Ras^V12^Dlg^RNAi^* vs *Ras^V12^Dlg^RNAi^* NAC P=0.06; *Ras^V12^Dlg^RNAi^SHMT^R^* vs *Ras^V12^Dlg^RNAi^SHMT^R^* dTMP P=0.73; *Ras^V12^Dlg^RNAi^SHMT^R^* vs *Ras^V12^Dlg^RNAi^SHMT^R^* NAC P=0.87.


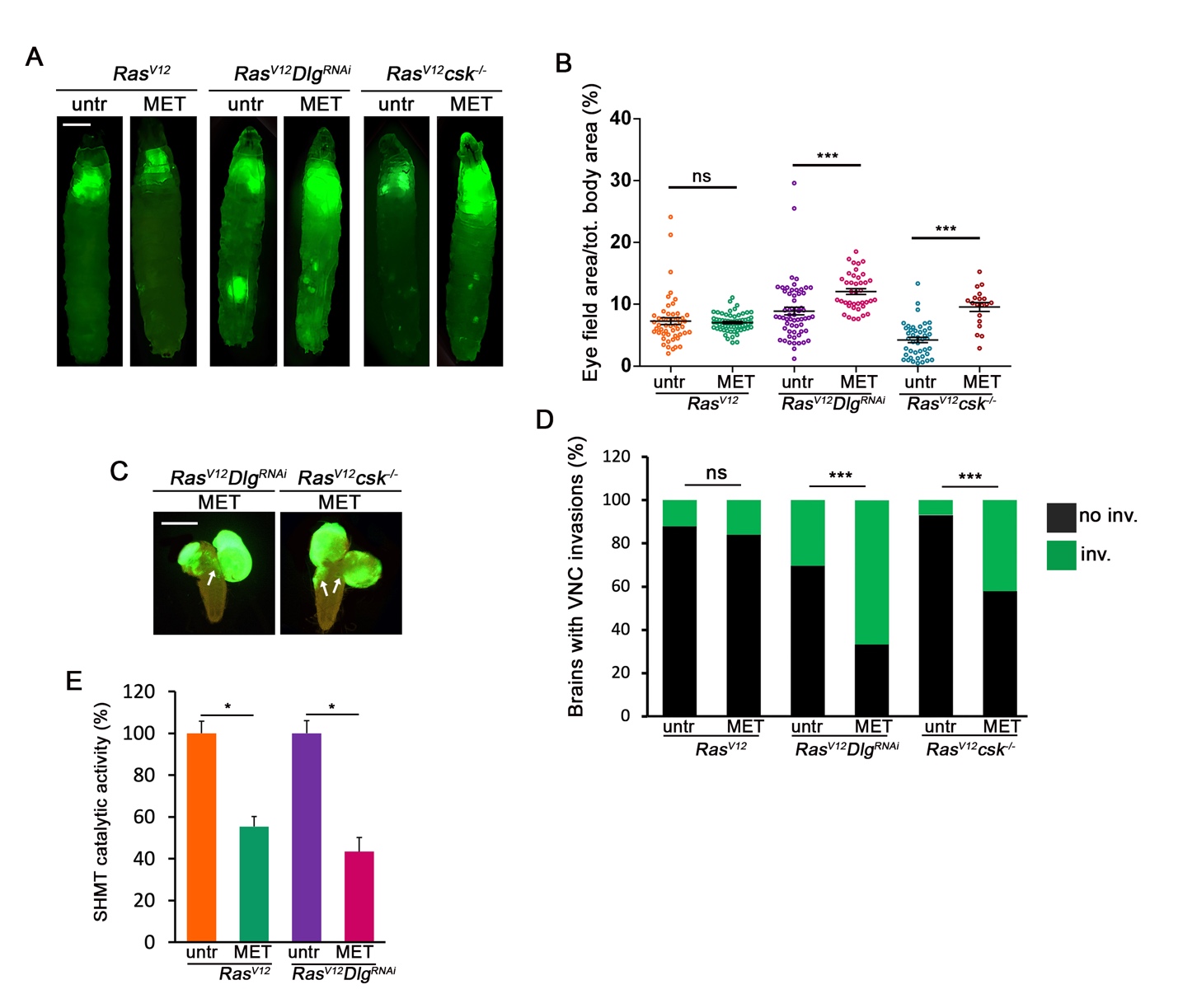


**Figure S6** SHMT inhibitor metformin (MET) increases cephalic area and VNC invasion frequency in *Ras^V12^Dlg^RNAi^* and *Ras^V12^csk^-/-^* models but fails to trigger the transformation of benign *Ras^V12^* tumors. **A** Representative image of GFP-labeled primary tumors in larvae reared on standard medium (untr) or standard medium supplemented with 50mM metformin (MET). Scale bar, 0.5mm. The *Ras^V12^* model was generated by expressing the oncogene *Ras^V12^* throughout the entire eye imaginal disc. *Ras^V12^ csk^-/-^* tumors were generated by *Mosaic analysis with a repressible cell marker* (MARCM) system, a strategy based on FLP-FRT mitotic recombination (1). This genetic technique yields cell clones which simultaneously express the oncogene *Ras^V12^* and carry a homozygous null mutation for *csk* (a negative regulator of Src kinase), surrounded by wild type cells. Note that *Ras^V12^csk^-/-^* cells typically undergo apoptosis (36), but MET treatment counteracts this trend leading to increased cell proliferation. **B** Quantification of GFP-positive eye field area relative to total body area (%). Error bars, SEM. ***P<0.001 (unpaired *t*-test). Ns=not significant *Ras^V12^* MET vs *Ras^V12^* P=0.65. Number of scored larvae in at least three independent experiments. *Ras^V12^ n=*51; *Ras^V12^* MET *n=*61; *Ras^V12^Dlg^RNAi^* *n=*60; *Ras^V12^Dlg^RNAi^* MET *n=*40; *Ras^V12^csk^-/-^ n=*43; *Ras^V12^csk^-/-^* MET *n=*19. **C** Larval brains showing tumor invasions on the ventral nerve cord (VNC), arrowed. Scale bar, 100μm. **D** Quantification of results. The green-labeled portion of each column represents the percentage of brains with VNC invasions. Statistics were assessed by chi square test ***P<0.001. Ns=not significant. *Ras^V12^* MET vs *Ras^V12^* P=0.55. Number of scored brains in at least three independent experiments: *Ras^V12^n=*49; *Ras^V12^* MET *n=*55; *Ras^V12^Dlg^RNAi^* *n=*46; *Ras^V12^Dlg^RNAi^* MET *n=*39; *Ras^V12^csk^-/-^ n=*43; *Ras^V12^csk^-/-^* MET n=19. **E** SHMT catalytic activity was measured in eye discs of indicated genotypes, both untreated and treated with 50mM MET. MET feeding effectively reaches the eye discs and reduces SHMT activity in both the *Ras^V12^* and *Ras^V12^Dlg^RNAi^* models. Error bars, SEM. *P<0.05 (unpaired *t*-test). The *Ras^V12^csk^-/-^* genotype was excluded from the assay due to critically low sample yield resulting from the FLP/FRT strategy, increased drug-induced mortality, and the clonal nature of the tumors. Note that metformin concentrations used here vastly exceed human therapeutic doses (2).


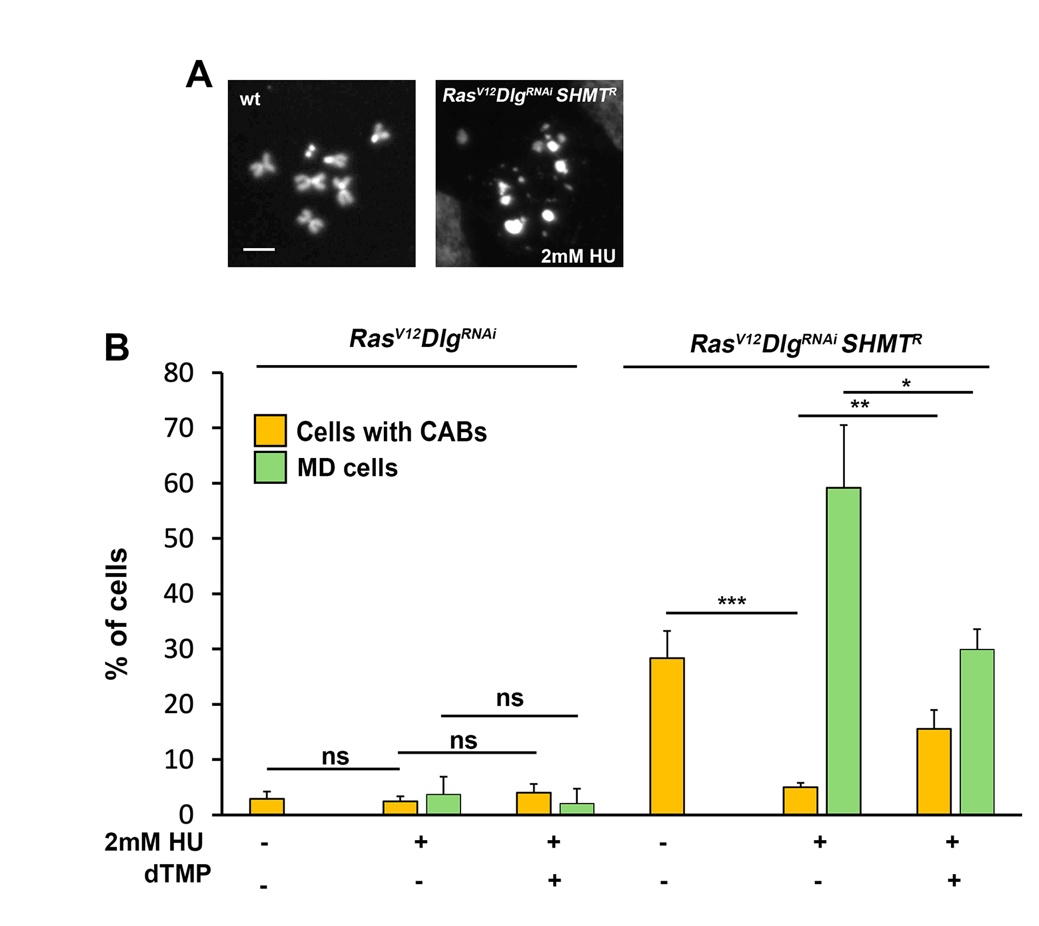


**Figure S7** 2mM HU treatment causes mitotic death (MD) and decreases the percentage of cells with CABs in *Ras^V12^Dlg^RNAi^ SHMT^R^* eye discs. **A** Example of MD cell with highly condensed chromosomes. Scale bar, 5μm. **B** Quantification of the results. dTMP supplementation increases the percentage of CABs in HU-treated cells and decreases the percentage of MD cells. Error bars, SEM. *P<0.05; **P<0.01; ***P<0.001 (unpaired *t*-test). ns=not significant. *Ras^V12^Dlg^RNAi^* untr vs HU-treated *Ras^V12^Dlg^RNAi^* % CABs P=0.794; HU-treated *Ras^V12^Dlg^RNAi^* vs HU+dTMP-treated *Ras^V12^Dlg^RNAi^* % CABs P=0.774, %MD P=0.66. Number of examined cells: *Ras^V12^Dlg^RNAi^ n=*450; HU-treated *Ras^V12^Dlg^RNAi^* *n=*904; HU+dTMP-treated *Ras^V12^Dlg^RNAi^ n=*299; *Ras^V12^Dlg^RNAi^ SHMT^R^ n=*505*;* HU-treated *Ras^V12^Dlg^RNAi^ SHMT^R^ n=*522; HU+dTMP-treated *Ras^V12^Dlg^RNAi^ SHMT^R^ n=*267*.*


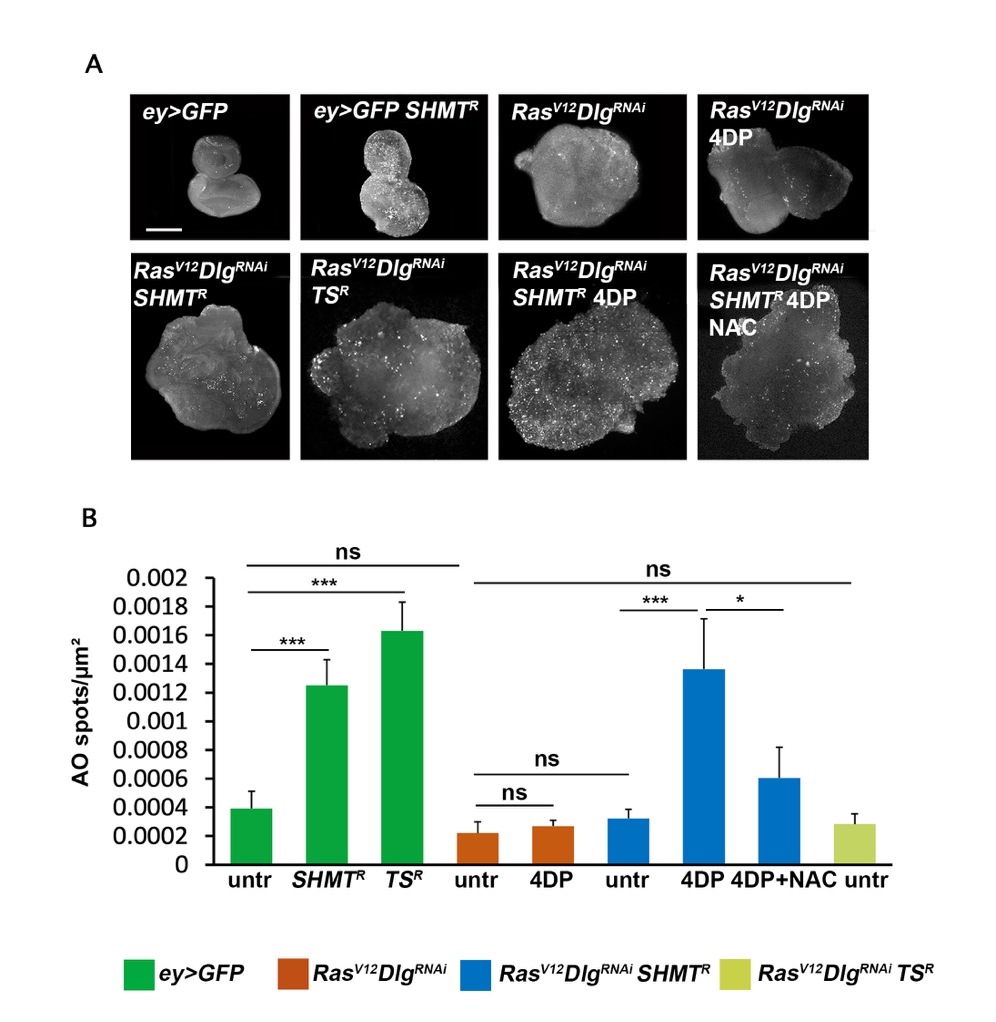


**Figure S8** **A** Eye discs of indicated genotypes and treatments stained with acridine orange (AO). Scale bar, 20μm. (NAC, 4mg/ml) **B** Quantification of results. AO spot density expressed as the number of AO-positive spots per square micrometer (μm^2^). Error bars, SEM. *P<0.05; ***P<0.001 (unpaired *t*-test). Ns=not significant *Ras^V12^Dlg^RNAi^* vs *ey>GFP* P=0.26; *Ras^V12^Dlg^RNAi^SHMT^R^* vs *Ras^V12^Dlg^RNAi^* P=0.76; *Ras^V12^Dlg^RNAi^* 4DP vs *Ras^V12^Dlg^RNAi^* P=0.42; *Ras^V12^Dlg^RNAi^TS^R^* vs *Ras^V12^Dlg^RNAi^* P=0.66. Number of scored discs in three independent experiments: *ey>GFP* *n*=16; *ey>GFP SHMT^R^* *n*=21; *ey>GFP TS^R^* *n*=15; *Ras^V12^Dlg^RNAi^* *n*=19; *Ras^V12^Dlg^RNAi^* 4DP *n*=16; *Ras^V12^Dlg^RNAi^SHMT^R^* *n*=17; *Ras^V12^Dlg^RNAi^SHMT^R^* 4DP *n*=20. *Ras^V12^Dlg^RNAi^SHMT^R^* 4DP NAC *n*=9; *Ras^V12^Dlg^RNAi^TS^R^* *n*=11.


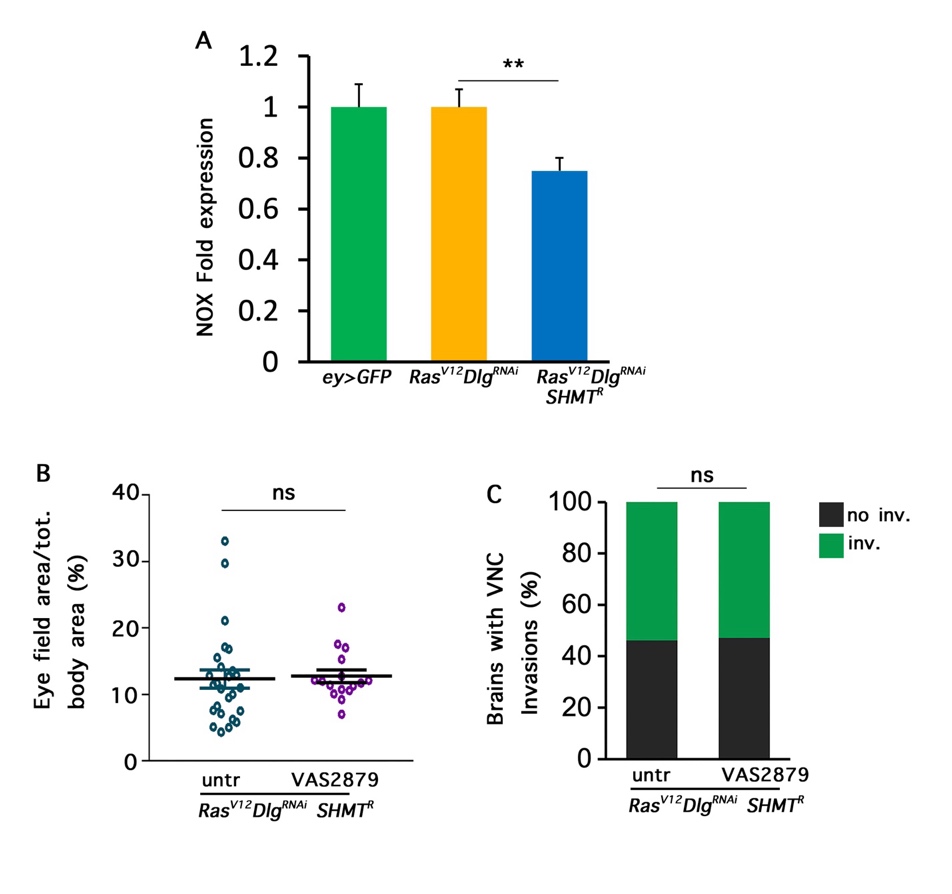


**Figure S9** ROS increase in *Ras^V12^Dlg^RNAi^SHMT^RNAi^* cells was not due to *NADPH oxidase (NOX)* overexpression. **A** Expression levels of *NOX* evaluated by RT-qPCR analysis in *Ras^V12^Dlg^RNAi^SHMT^RNAi^* eye discs. Unlike human cells depleted of dTMP (19)(40), NOX levels were found to be decreased rather than increased. Error bars, SEM. **P<0.01 (unpaired *t*-test). **B**, **C** Tumor phenotypes were unaffected by treatment with NOX inhibitor VAS2879. **B** Quantification of GFP-positive eye field area relative to total body area (%). Error bars, SEM. Ns=not significant (unpaired *t*-test) P=0.59. Number of scored larvae: *Ras^V12^Dlg^RNAi^SHMT^RNAi^* *n=*26; *Ras^V12^Dlg^RNAi^SHMT^RNAi^* VAS2879 *n=*17. **C** VNC invasion quantification. Statistics were assessed by chi square test. Ns=not significant P=0.95. Number of scored brains: *Ras^V12^Dlg^RNAi^SHMT^RNAi^* *n=*26; *Ras^V12^Dlg^RNAi^SHMT^RNAi^* VAS2879 *n=*17.

**References**

1. Lee T, Luo L. Mosaic analysis with a repressible neurotechnique cell marker for studies of gene function in neuronal morphogenesis. Neuron. 1999;22:451–61.
2. Umekar M, Qutub M, Premchandani T, Tatode A,Taksansde J, Singanwad P, et al. Molecular aspects of metformin’s anti-aging properties for muscle function and longevity in Drosophila melanogaster. Precision Medication 2025;2:100051.
